# Spatiotemporal transcriptomic maps of whole mouse embryos at the onset of organogenesis

**DOI:** 10.1038/s41588-023-01435-6

**Published:** 2023-07-06

**Authors:** Abhishek Sampath Kumar, Luyi Tian, Adriano Bolondi, Amèlia Aragonés Hernández, Robert Stickels, Helene Kretzmer, Evan Murray, Lars Wittler, Maria Walther, Gabriel Barakat, Leah Haut, Yechiel Elkabetz, Evan Z. Macosko, Léo Guignard, Fei Chen, Alexander Meissner

**Affiliations:** 1 Department of Genome Regulation, Max Planck Institute for Molecular Genetics, Berlin, Germany; 2 Institute of Biotechnology, Technische Universität Berlin, Berlin, Germany; 3 Broad Institute of MIT and Harvard, Cambridge, MA USA; 4 Institute of Chemistry and Biochemistry, Freie Universität Berlin, Berlin, Germany; 5 Graduate School of Arts and Sciences, Harvard University, Cambridge, MA USA; 6 Department of Developmental Genetics, Max Planck Institute for Molecular Genetics, Berlin, Germany; 7 Department of Psychiatry, Massachusetts General Hospital, Boston, MA USA; 8 Aix Marseille University, Toulon University, Centre National de la Recherche Scientifique, Laboratoire d’Informatique et Systèmes 7020, Turing Centre for Living Systems, Marseille, France; 9 Department of Stem Cell and Regenerative Biology, Harvard University, Cambridge, MA USA

**Keywords:** Embryogenesis, Transcriptomics

## Abstract

Spatiotemporal orchestration of gene expression is required for proper embryonic development. The use of single-cell technologies has begun to provide improved resolution of early regulatory dynamics, including detailed molecular definitions of most cell states during mouse embryogenesis. Here we used Slide-seq to build spatial transcriptomic maps of complete embryonic day (E) 8.5 and E9.0, and partial E9.5 embryos. To support their utility, we developed sc3D, a tool for reconstructing and exploring three-dimensional ‘virtual embryos’, which enables the quantitative investigation of regionalized gene expression patterns. Our measurements along the main embryonic axes of the developing neural tube revealed several previously unannotated genes with distinct spatial patterns. We also characterized the conflicting transcriptional identity of ‘ectopic’ neural tubes that emerge in *Tbx6* mutant embryos. Taken together, we present an experimental and computational framework for the spatiotemporal investigation of whole embryonic structures and mutant phenotypes.

## Abstract (web summary)

Slide-seq profiling of mouse embryos at the onset of organogenesis (embryonic days 8.5–9.5) coupled with a new three-dimensional reconstruction and visualization tool (sc3D) provides high-resolution maps of spatiotemporal gene expression dynamics.

## Main

Embryonic development necessitates the precise timing and location of numerous molecular, cellular and tissue-level processes^1–5^. These events are directed via spatiotemporal control of gene expression that orchestrates cell type specification, migration and localization^6–9^. Any disruption of this regulation often results in embryonic lethality or developmental defects^5,10,11^. At the end of gastrulation and the onset of organogenesis (embryonic days (E) 8.5–9.5), tissues experience major morphological changes, such as heart looping, brain compartmentalization and neural tube folding, to guarantee proper structure and function^12–14^. Through neurulation, epithelial cells in the neural plate fold to form a morphologically defined tube, which exhibits a stratified gene expression signature along its dorsoventral (DV) axis, which is necessary for subsequent neuronal subtype diversification^15–21^. Many genes involved in this process have been identified, but the precise gene regulatory networks governing these patterns remain under investigation. Recent single-cell studies have begun to provide a deeper understanding of the topography of fate specification and highlighted some molecular mechanisms underlying these cell state transitions^22–28^. One limitation of dissociation-based approaches is their inability to preserve tissue structure, which precludes expression analysis within the native context. Recent advances in spatial transcriptomic technologies have begun to fill this gap, aiming to explore the organization of cell types within adult tissues and developing embryos^29–38^.

In this study, we used Slide-seq, a technology that generates transcriptome-wide gene expression data at 10-µm spatial resolution^33,39^, to build maps of whole embryos during early mouse organogenesis. Our data enabled the exploration of spatial gene expression patterns, cell state distributions, the reconstruction of three-dimensional (3D) transcriptomic maps for ‘virtual’ gene expression analysis and mutant phenotype dissection. We specifically leveraged the data to identify regionalized gene expression and differentiation trajectories in space, focusing on neural tube formation and patterning. Overall, we provide a comprehensive, high-resolution spatial atlas together with an accessible and ready-to-use visualizer to explore gene expression patterns in the developing mouse embryo.

## Results

### Spatial transcriptomic maps to construct 3D virtual embryos

**Fig. 1:**
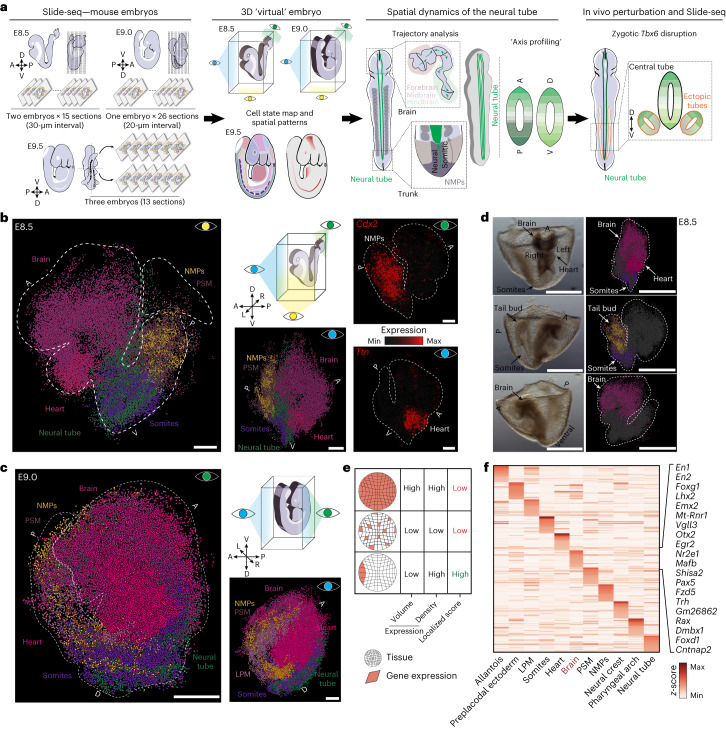
Embryo-wide profiling of gene expression with spatial coordinates using Slide-seq. **a**, Schematic of the experimental workflow and data analysis. Sagittal sections of mouse embryos at E8.5, E9.0 and E9.5 were obtained for Slide-seq. The dotted lines indicate the approximate position of the embryonic sections. **b**,**c**, 3D-reconstructed E8.5 (**b**) and E9.0 (**c**) stage embryos with six cell states highlighted (brain, heart, neural tube, somites, NMPs, PSM); the caudal marker gene *Cdx2* and heart tube marker gene *Ttn* are shown (normalized gene expression) in a vISH of the reconstructed E8.5 embryo1. Each dot corresponds to a single bead. Point of view is denoted by the eye symbol. Scale bars, 100 µm. **d**, 3D view of an E8.5 embryo showing the indicated cell states and anatomical features in brightfield (left) or mapped onto the E8.5 embryo1 (right). Different orientations are displayed. Scale bars, 500 µm. **e**, Schematic of the strategy used to identify localized gene expression in tissues. **f**, Heatmap of localization scores for the top 20 spatially differentially expressed genes in 3D for each analyzed tissue in the E8.5 embryo1 (see Supplementary Table 4 for the list). Top 20 genes (row *z*-score-normalized) in the brain are highlighted. A, anterior; LPM, lateral plate mesoderm; P, posterior; D, dorsal; V, ventral; LPM, lateral plate mesoderm; NMPs, neuromesodermal progenitors; PSM, pre-somitic mesoderm.

**Extended Data Fig. 1:**
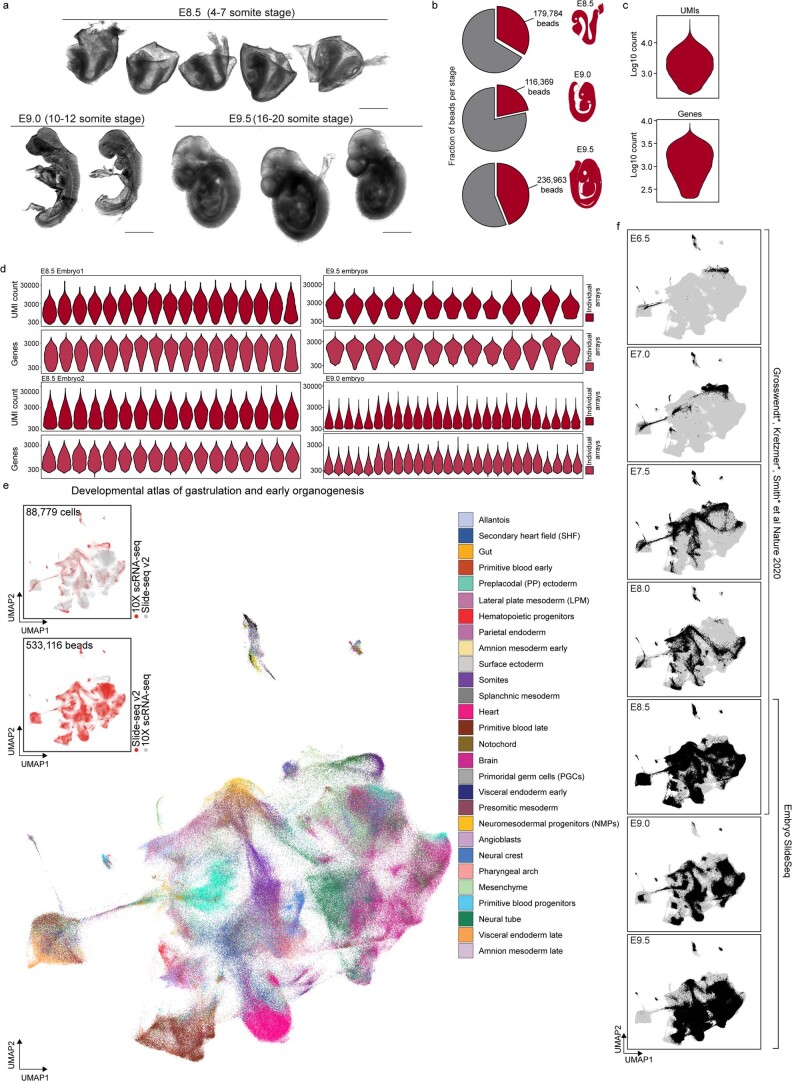
Integration and cell-type annotation. **a**. Brightfield images of representative embryos isolated from 3 independent foster mice and staged for the respective developmental stages. Scale bar, 500 µm. **b**. Distribution of beads profiled by Slide-seq from the respective stages. **c**. Violin plots showing the number of UMIs or genes recovered per bead. Log_10_ values are used to represent counts. UMI, unique molecular identifier. **d**. Violin plots showing the number of UMIs and genes recovered per bead across individual arrays. Log_10_ values are used to represent counts. UMI, unique molecular identifier. **e**. Uniform Manifold Approximation and Projection (UMAP) of Slide-seq data and 10X scRNA-seq reference atlas of mouse gastrulation^26^. The color of the beads corresponds to the predicted and annotated cell state. Inset: UMAP representation of beads covered by the indicated modalities (red). Each dot represents a bead or a cell. **f**. UMAP of integrated data from stages E6.5 to E9.5. Black beads represent cells/beads from the corresponding stage. Each dot represents a bead or a cell.

**Extended Data Fig. 2:**
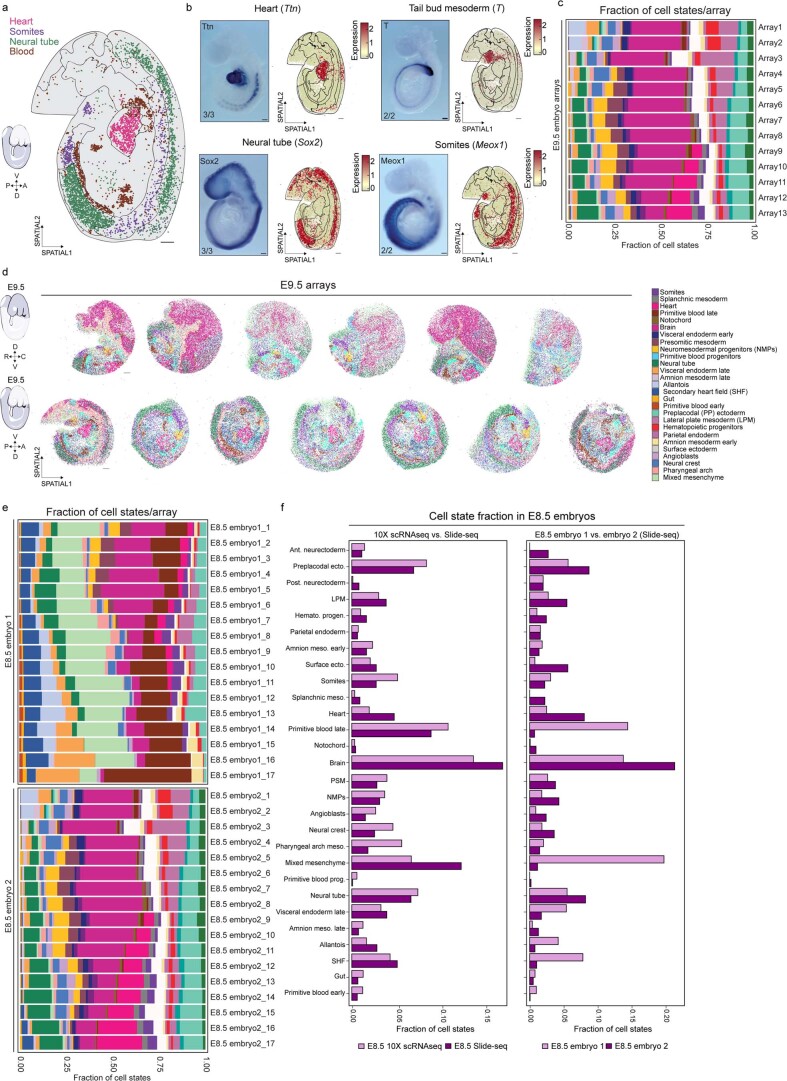
Spatial organization of cell states. **a**. Representative trunk/thoracic array (array E9.5_2) with highlighted cell states projected spatially. Each dot represents a bead. Outlines are used to emphasize morphological characteristics. A, anterior; P, posterior; D, dorsal; V, ventral. Scale bar, 200 μm **b**. Comparison of Slide-seq and conventional whole-mount *in situ* hybridization for gene expression patterns. Spatial gene expression plot showing the expression of *Ttn* (heart), *Sox2* (neural tube), *T* (tailbud mesoderm), and *Meox1* (somites). The color scale depicts normalized gene expression. n/n in the whole-mount *in situ* hybridization panel indicates the number of embryos exhibiting the pattern to the total number of embryos assayed (from one experiment). Each dot represents a bead. Scale bar, 200 μm. **c**. Cell states distribution of annotated clusters in the individual E9.5 arrays. Colors represent individual cell states, legend in panel (**d**). **d**. Spatial projection of annotated cell states in E9.5 embryo arrays. The panel depicts arrays that cover the trunk/thoracic and head regions. Each color corresponds to a distinct cell state. Each dot represents a bead. Scale bar, 200 μm. A, anterior; P, posterior; D, dorsal; V, ventral; R, rostral; C, caudal. **e**. Cell state distribution of the annotated clusters in individual arrays of the two independently profiled whole E8.5 stage embryos. Colors represent individual cell states according to the legend in panel (**d**). **f**. Cell state distribution of individual states in 10X scRNA-seq reference^26^ and Slide-seq at E8.5 stage (left panel), and the comparison between the two whole E8.5 embryos profiled by Slide-seq (right panel).

To spatially map cell identities during early organogenesis, we used Slide-seq on two representative E8.5, one E9.0 and three partial E9.5 embryos (Fig. 1a and Extended Data Fig. 1a). For the two E8.5 embryos, we obtained 15 and 17 sagittal sections (10-µm thickness), respectively, with approximately 30-µm intervals between them. For the E9.0 embryo, 26 sagittal sections with 20-µm intervals, and for the three E9.5 embryos, 13 slices from the midline were obtained (Fig. 1a and Supplementary Table 1). In total, we recovered 533,116 high-quality beads with a median value of 1,798 transcripts and 1,224 genes per bead (Extended Data Fig. 1b–d). To ascertain the cell states assigned to each bead, we computationally mapped beads to a previously generated single-cell reference (Extended Data Fig. 1e,f)^26^. With this information, we extracted from each sequenced bead (1) spatial coordinates, (2) associated gene expression profile and (3) cell state assignment. Overall, we observed good alignment of cell states and spatial restriction of marker genes, such as *Ttn* (heart), *T* (tail bud), *Meox1* (somites) and *Sox2* (neural tube, brain), among others (Extended Data Fig. 2a–e). Additionally, we observed high reproducibility in recovering a comparable embryo composition and gene expression patterns among replicates (Extended Data Fig. 2c–f).

**Extended Data Fig. 3:**
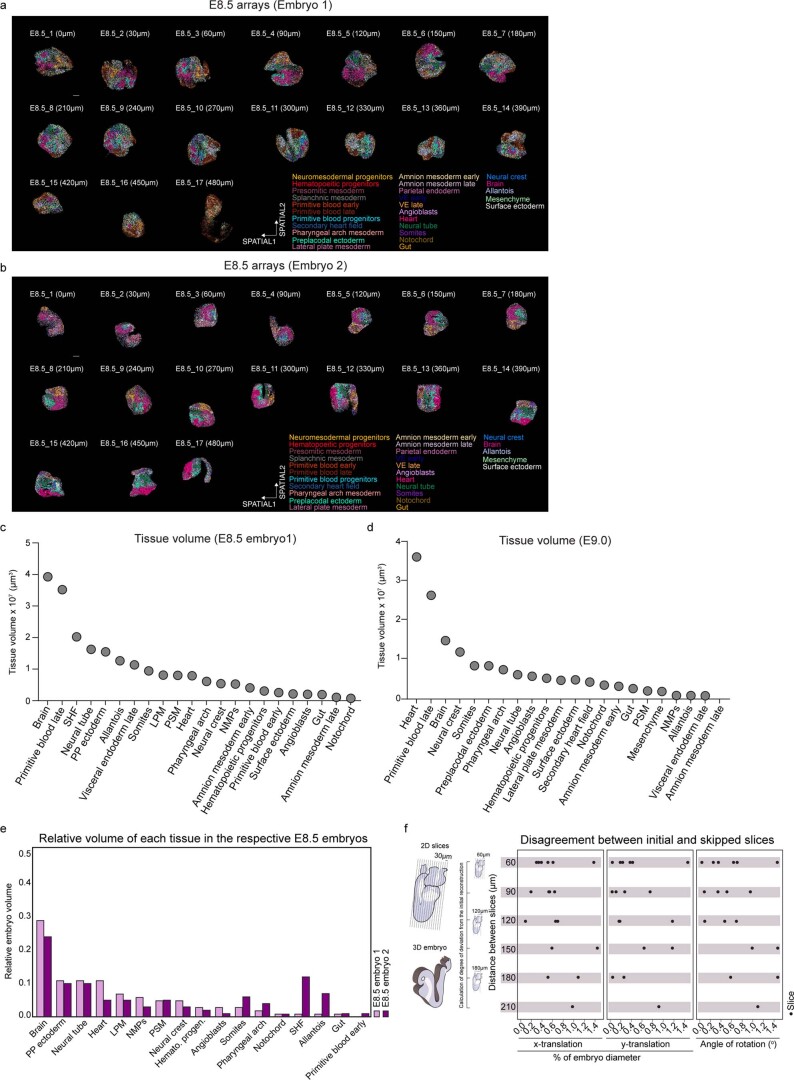
E8.5 and E9.0 3D embryos. **a**, **b**. Spatial projection of cell states in individual arrays of the two whole E8.5 embryos. Each dot corresponds to a bead. Each color represents a cell state. Scale bar, 200 μm. **c-d**. Volumes of the indicated tissues ranked from the largest to the smallest, calculated from the E8.5 and E9.0 3D virtual embryo. Tissue volumes are listed in Supplementary Table 2. **e**. Relative volume of each tissue in the two whole E8.5 embryos. **f**. Disagreement between the initial and skipped slices when increasing the distance between individual slices.

**Extended Data Fig. 4:**
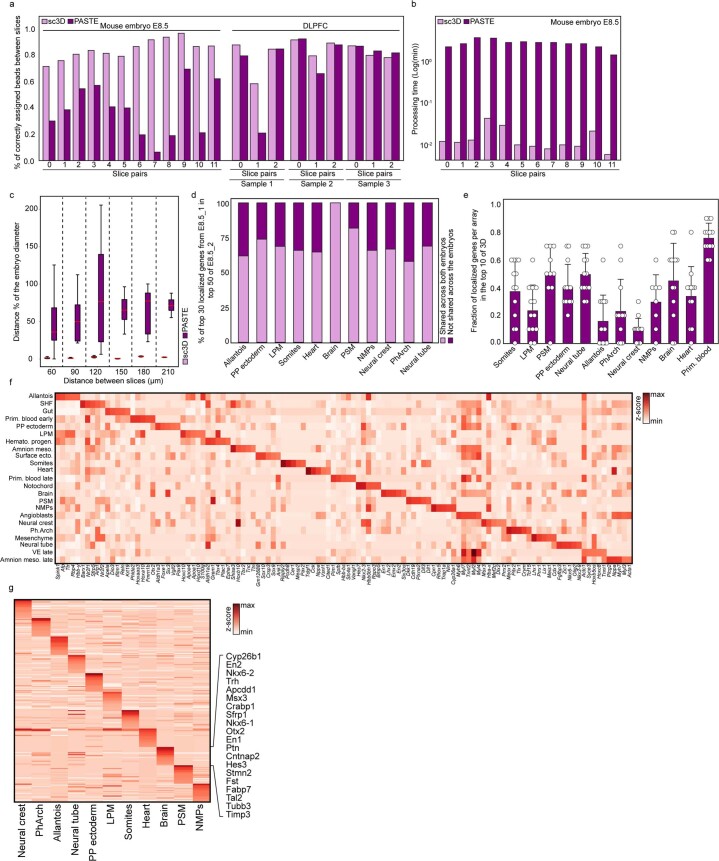
Robustness of sc3D. **a**, **b**. Comparison of accuracy and processing time (*sc3D* vs PASTE) across different datasets. **c**. Boxplot of the pairwise distances between the beads in the correctly registered images and those generated by the algorithm when slices are separated by 60, 90, 120, 150, 180, 210 µm (‘n’ slice pairs: 6,4,3,3,2,1, respectively). Boxplot elements: middle line, median; box plot limits, upper and lower quartiles; whiskers, standard deviation. **d**. Barplot of the overlap of localized genes across tissues in the two individual E8.5 whole embryos. Complete list of genes can be found in Supplementary Table 3. **e**. Barplot of the fraction of localized genes in the individual 2D arrays that were in the top 10 ranked localized genes in 3D volume of the respective tissues across all tissues in the E8.5 embryo (shown is the analysis for E8.5_Embryo_1). Error bars denote standard deviation of genes between the 2D slices and 3D volume (n = 13 genes). **f-g**. Heatmap of top localized genes (row z-score normalization) across the indicated tissues in the E8.5 embryo_1 (**f**) and E9.0 (**g**) embryo.

To translate our two-dimensional data into a 3D embryo, we developed sc3D, a computational method that enables the alignment of individual spatial transcriptomic arrays for 3D reconstruction. Specifically, we used sc3D to align serial Slide-seq ‘*z*’ samples from E8.5 and E9.0 embryos, which allowed us to capture the spatial distribution and morphologies of the emerging tissues at the onset of organogenesis (Extended Data Fig. 3a,b and Supplementary Figs. 1 and 2). This in turn enabled quantitative measurements of their volumes (250–39,264 × 10^3^ µm^3^), which were reproducible across individual replicate embryos (Extended Data Fig. 3c–e, Supplementary Fig. 1, Supplementary Movie 1 and Supplementary Table 2). We further showed that the reconstruction remained consistent as the interval between slices increased, with very minimal distortion in the rotation axes (Extended Data Fig. 3f). Importantly, sc3D also allows 3D reconstruction of other spatial transcriptomic datasets with high precision, speed and robustness to reduced spatial resolution^40^ (Extended Data Fig. 4a–c and Supplementary Table 3).

**Fig. 2:**
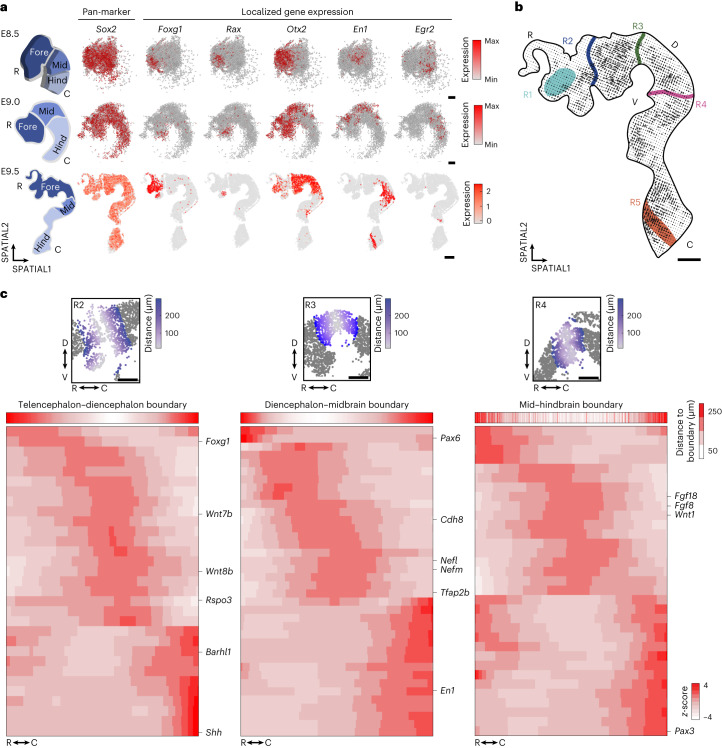
Localized gene expression patterns in the developing brain. **a**, Schematic (left) and normalized gene expression spatial plot (right) of selected genes (highlighted in Fig. 1f) in the 3D brain of E8.5 embryo1, E9.0 and E9.5 (array E9.5_3). Each dot represents a single bead. **b**, Spatial plot of RNA velocity in the E9.5 (array E9.5_3) brain region. Vector direction indicates the trajectory and length denotes the magnitude. Low-velocity regions are indicated as R1, R2, R3 and R4. R1, neural ridge prosencephalon; R2, telencephalon–diencephalon boundary; R3, diencephalon–mesencephalon boundary; R4: mesencephalon–hindbrain boundary; and R5, hindbrain–spinal cord boundary. **c**, Spatial plot showing the brain boundaries (top) and top enriched spatially differentially expressed genes along the R2, R3 and R4 brain boundaries (represented as row *z*-score-normalized expression) at E9.5 (array E9.5_3). The full list of genes can be found in Supplementary Table 5. Scale bar for all plots, 50 µm. C, caudal; D, dorsal; R, rostral; V, ventral.

**Extended Data Fig. 5:**
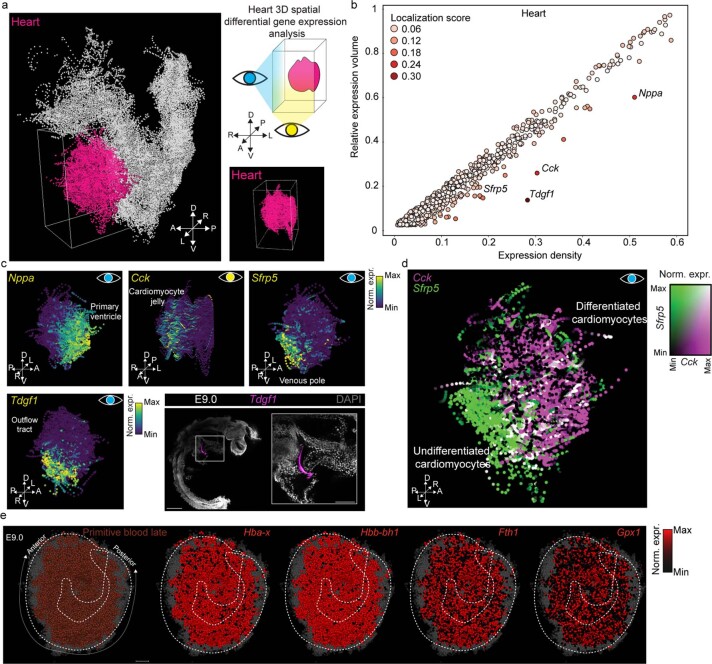
Localized gene expression in the developing heart. **a**. 3D view of E8.5 embryo highlighting the heart tissue volume (in pink). Each dot corresponds to a bead. The point of view is denoted by the eye symbol (yellow or blue). **b**. Scatter plot showing the top localized genes in the heart tissue volume. Color scale corresponds to localization score ranging from 0.06 (light red) to 0.30 (dark red). Each dot represents a gene. Spatial localization of the highlighted genes is displayed in **c**. *x-*axis shows the density of gene expression, and *y-*axis shows the relative volume of expression within the tissue. **c**. vISH of localized genes, *Nppa, Cck, Sfrp5 and Tdgf1*. Color scale denotes the normalized gene expression values ranging from minimum to maximum for every gene. Each dot corresponds to a bead. The point of view is denoted by the eye symbol (yellow or blue). RNA-FISH in the E9.0 embryo shows the distinct localization of *Tdgf1* in the heart. Scale bar, 100 µm. **d**. vISH showing gene co-expression for *Cck* (magenta) and *Sfrp5* (green) in the heart tissue. Each dot corresponds to a bead. Color scale for each gene ranges from black to magenta, or black to green. Beads double-positive are displayed in white. **e**. vISH for spatially ubiquitous genes in the state ‘primitive blood late’. Scale bar, 200 µm. A, anterior; P, posterior; D, dorsal; V, ventral; L, left; R, right.

We next used the reconstructed embryos to perform ‘virtual’ in situ hybridization (vISH) of over 27,000 genes on a quantitative scale, with the opportunity to query gradient gene expression along any given body axis, inclination plane and rotation angle (Fig. 1b–d, Supplementary Figs. 1 and 2, and Supplementary Movies 1–4). To further increase sc3D accessibility, we developed sc3D-viewer, a user-friendly and interactive environment for exploring the Uniform Manifold Approximation and Projection (UMAP) representation, spatial cell state maps, and vISH for single and dual gene combinations (methods in Supplementary Information). To investigate spatial gene expression within each tissue type, we computed the genome-wide correlations between tissue volumes and densities of expressing cells to generate a localization score that allowed us to rank genes within each tissue based on their spatial restriction in expression. This analysis identified a set of highly informative, tissue-specific, regionalized genes within the embryo and across replicates as well as developmental stages (Figs. 1e,f and 2a, Extended Data Fig. 4d–g and Supplementary Table 4). For instance, we found high localization scores for genes such as *Nppa*, *Tdgf1*, *Cck* and *Sfrp5* in the developing heart tube (Extended Data Fig. 5a–d and Supplementary Movie 5). Mapping these genes onto our digital embryo revealed that their expression domains mark specific developmental axes (anterior–posterior (AP), DV and right-left) and delineate presumptive anatomical structures, such as the primitive ventricles and atria, the outflow tract, the cardiomyocyte jelly and the venous pole, respectively^41–43^, as opposed to spatially ubiquitous gene expression seen in the blood (Extended Data Fig. 5c–e). Furthermore, *Cck* and *Sfrp5* expression spatially distinguish differentiated from undifferentiated cardiomyocytes domains (Extended Data Fig. 5d)^41–43^. Taken together, this demonstrates our ability to identify regionalized markers and study the distinct domain organization within complex developing tissues along developmental axes.

### Delineating molecular boundaries in the developing brain

**Extended Data Fig. 6:**
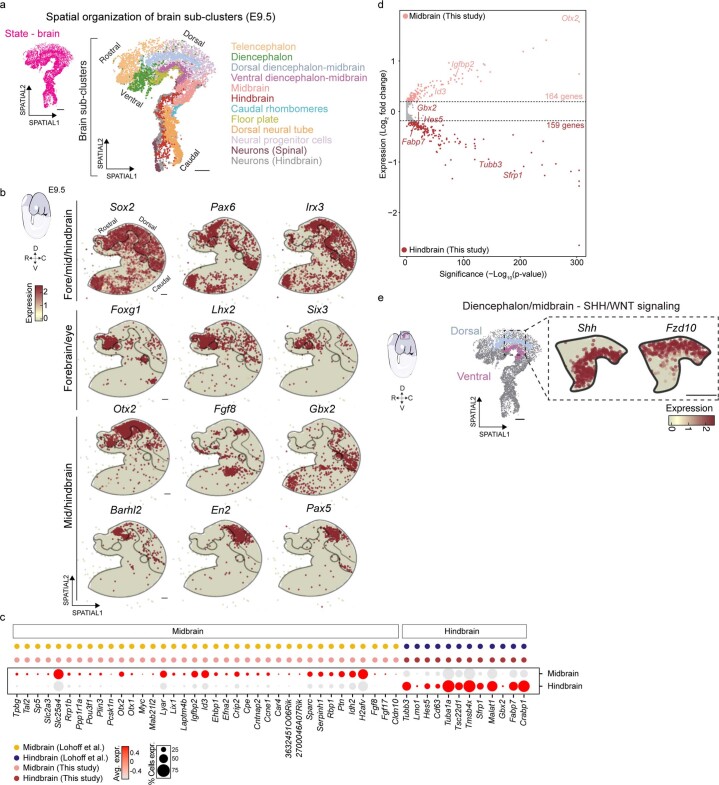
Spatial organization in the developing brain. **a**. Spatial plot of the annotated clusters/states in the brain region of an E9.5 stage embryo (array E9.5_5). Each dot corresponds to a bead. Each color represents the annotated cell state. Scale bar, 200 µm. **b**. Spatial gene expression plot depicting the indicated genes of the mentioned categories. Each dot represents a bead. The color scale depicts normalized gene expression. Scale bar, 200 µm. Array shown is E9.5_3. **c**. Dotplot showing the expression of mid and hindbrain genes (annotated^25^ in the mid and hindbrain clusters in this study). Size of the dot represents the percentage of cells expressing the genes, and the color denotes the normalized gene expression. **d**. Volcano plot of the differentially expressed genes between the annotated mid and hindbrain (this study). Each dot represents a gene, dots in grey are below the significance threshold (genes filtered by FDR < 0.01 and logFC>0.2; Two-sided Wilcoxon ran sum test). **e**. Schematic and spatial expression plot distinguishing dorsal and ventral diencephalon/midbrain regions. *Shh* marks the ventral domain, whereas *Fzd10* (Wnt receptor) marks the dorsal part. Each dot represents a bead. The color scale depicts normalized gene expression. Scale bar, 100 µm. D, dorsal; V, ventral; R, rostral; C, caudal.

**Extended Data Fig. 7:**
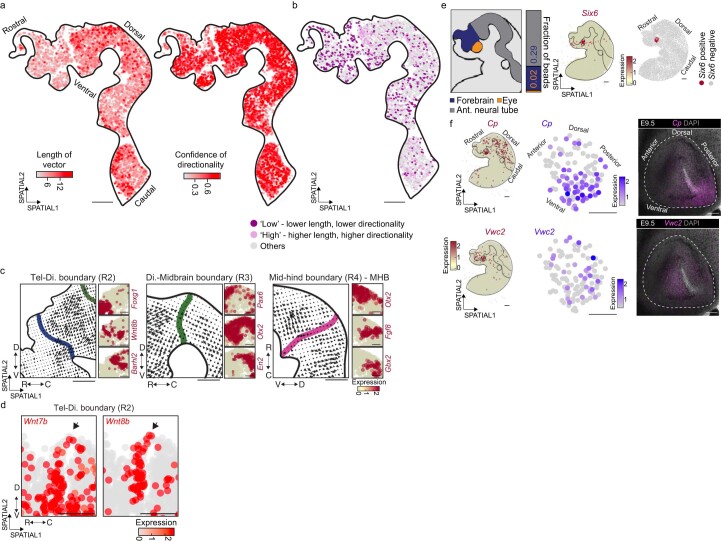
Patterning of the developing brain. **a**. Spatial velocity length (left) and confidence of directionality (right) highlight regions with different dynamics. Scale bar, 200 µm. Array shown is E9.5_3. **b**. Combining the length and confidence measurement results in ‘low’ and ‘high’ velocity regions. Regions with ‘low’ velocity display lower length and lower directionality. Regions with ‘high’ velocity display higher length and directionality of the vector. Scale bar is 200 *µm*. Array shown is E9.5_3. **c**. Inset regions of the RNA velocity in the brain region with expression of markers defining the respective boundary regions (representative of 3 independent E9.5 arrays). Each dot corresponds to a bead. Color scale denotes normalized expression. Scale bar, 50 µm. **d**. Spatial plot of WNT genes at R2 (Telencephalon-diencephalon boundary – denoted arrows; representative of 3 independent E9.5 arrays). Each dot corresponds to a bead. Color scale denotes normalized expression. Scale bar, 50 µm. **e**. Slide-seq based schematic of the developing eye. The forebrain (dark blue) and the eye (orange) are depicted with the fraction of beads corresponding to each state (bar plot). 1499/4824 beads for the forebrain/anterior neural tube; 105/4824 beads for the eye/anterior neural tube; and 105/1499 beads for the eye/forebrain. Spatial plot showing the expression of the eye-specific marker *Six6*. A, anterior; R, rostral; C, caudal; D, dorsal; V, ventral. **f**. Spatial plots showing the expression of two newly identified eye marker genes, *Cp* and *Vwc2* (left: whole E9.5_3 array; middle: subset of the ‘eye’; right: RNA-FISH validations for *Cp* and *Vwc2* (magenta) and counterstained nuclei (grey) are shown for a representative embryo. n = 3 embryos/experiment, 3 independent experiments). Scalebar, 200 µm. R, rostral; C, caudal; D, dorsal; V, ventral.

Between E8.5 and E9.5, the most anterior portion of the neural tube develops into three distinct vesicles (prosencephalon–forebrain, mesencephalon–midbrain and rhombencephalon–hindbrain), which together form the primordial brain^20,21,44–47^. Our high-resolution spatial transcriptomic map of the forebrain–prosencephalon in E9.5 embryos allowed us to identify presumptive telencephalon and diencephalon regions and to delineate DV patterning of the diencephalon–midbrain (Extended Data Fig. 6a–e)^48^. To study the emergence of such patterns, we analyzed the transcriptome of E8.5, E9.0 and E9.5 brains and found that genes such as *Foxg1*, *Barhl2*, *Otx2*, *En1* and *Egr2* show regionalized expression patterns already at E8.5 (Figs. 1f and 2a). While *Foxg1* was confined to the rostral prosencephalon, defining the presumptive telencephalon, *Barhl2* was expressed caudally, already marking the presumptive diencephalon^49–51^. *Rax*, a marker of the developing eye, exhibited spatial gene expression confinement between E8.5 and E9.5, defining the future optic cup (Fig. 2a). It appears therefore that spatial restriction of gene expression precedes anatomical segregation. To further explore the relationship between cell fate commitment and spatial restriction of emerging structures, we used unsupervised spatial RNA velocity without prior knowledge of cell states^52^. We recovered distinct ranges of velocity dynamics with either converging or diverging trajectories, potentially corresponding to stepwise transitions or cellular steady states (Fig. 2b and Extended Data Fig. 7a,b). A closer inspection into low-velocity regions (defined by the low-velocity length and confidence of vector directionality), combined with the expression of known marker genes, revealed the presence of progenitor field domains (R1 anterior neural ridge) as well as differentiated neuronal territories (R5 hindbrain–spinal cord boundary) (Extended Data Figs. 6b,c and 7c). Additionally, areas with diverging trajectories highlighted boundary regions, such as the R4 mesencephalon–rhombencephalon boundary, which was marked by the restrictive and exclusive expression patterns of *Otx2* in the mesencephalon and *Gbx2* in the rhombencephalon, as well as *Fgf8* at the boundary (Extended Data Fig. 7c)^53^.

Although vector ends do not necessarily represent a terminally differentiated state, such a relationship might be inferred when spatial trajectories are known to match with developmental patterning processes. For instance, the trajectories observed at R4 resemble the lineage specification of the mid–hindbrain progenitors that will generate neuronal cells that subsequently populate the whole midbrain and anterior hindbrain areas^21^. In addition, our high-resolution map enabled a more granular view of the molecular determinants of developing boundaries before the formation of anatomical constriction (Fig. 2c and Supplementary Table 5). We analyzed the three boundaries demarcating the main brain regions to identify features that might shed light on the regulatory mechanisms involved in brain regionalization. In R2 and R4, we identified several signaling molecules (WNT, FGF) and downstream effectors ratifying the role of these boundaries as signaling centers instructing the patterning of the adjacent structures (the zona limitans intrathalamica (ZLI) and the isthmic organizer). While the interplay between WNT and FGF signaling in the mid–hindbrain boundary (R4) is well known, their role at the telencephalon–diencephalon boundary (R2), where SHH signaling has a major role^54^, has been less studied. In this study, we show the spatial constriction of *Wnt7b* expression to the boundary together with *Wnt8b* (Extended Data Fig. 7d). Although *Wnt7b* is expressed in the rostral and dorsal parts of the diencephalon^55^, its co-expression and colocalization with *Wnt8b*, known to be expressed within the ZLI^56^, has not been described yet. Compared to the other analyzed regions, the diencephalon–midbrain boundary (R3) is characterized by low signaling molecule activity, high neuronal marker expression (neurofilament proteins and neuronal genes) and convergent RNA velocity signature, suggesting the presence of a more mature neuronal rather than a progenitor domain (Fig. 2c and Supplementary Table 5). Therefore, our analysis can help identify relevant molecules such as CDH8 (Fig. 2c and Supplementary Table 5), which is known to compartmentalize the diencephalon–midbrain boundary together with other cadherins^57^. Moreover, within the forebrain region, we mapped known as well as uncharacterized gene expression distributions, including those involved in eye development (Extended Data Fig. 7e,f). By examining such patterns during early brain development, we stratified the spatial emergence of anatomical structures.

### Cell identity and spatial distance in the trunk

**Fig. 3:**
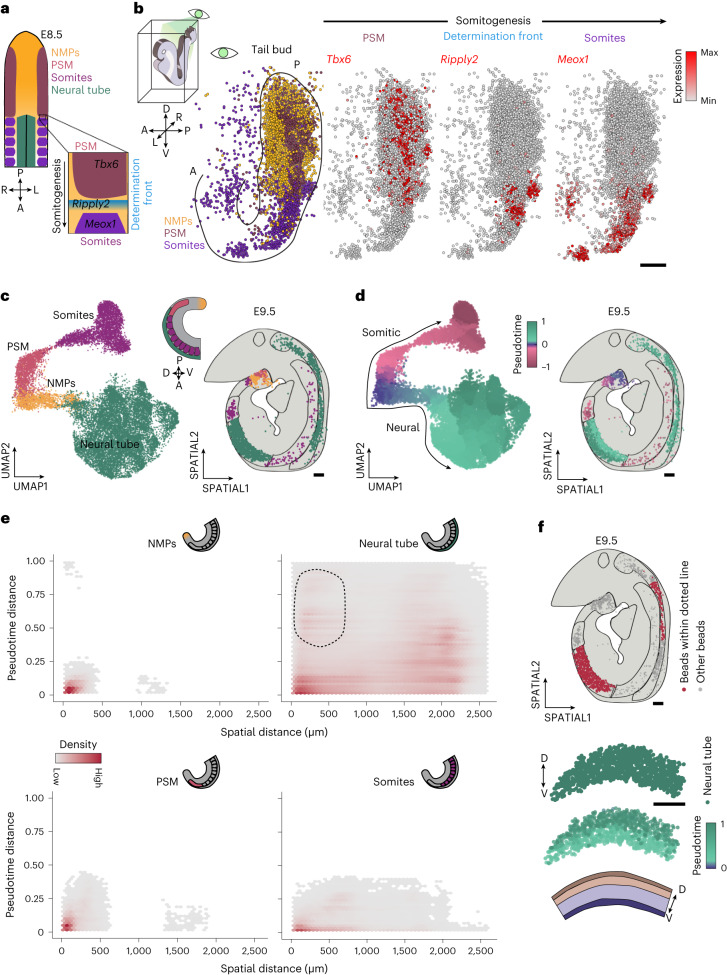
Spatial organization of the embryonic trunk. **a**, Schematic of the spatial organization of cell states in the embryonic trunk region at E8.5, and close-up view on the somitogenesis process. **b**, Schematic and 3D spatial plot of E8.5 (embryo2), showing selected cell states and vISH of *Tbx6*, *Ripply2* and *Meox1* in the trunk region. Each dot denotes a bead and the color corresponds to the indicated state. Scale bar, 200 µm. **c**, UMAP showing beads from E8.5 and E9.5 embryos corresponding to the NMPs, PSM, somites and neural tube states (left) and the corresponding spatial distribution (right) in an E9.5 embryo (array E9.5_2). Each dot denotes a bead and the color corresponds to the indicated state. Scale bar, 100 µm. **d**, UMAP showing pseudotime analysis along the somitic and neural differentiation trajectories (left) and the corresponding spatial distribution (right) in an E9.5 embryo (array E9.5_2). Each dot denotes a bead and the color corresponds to the assigned pseudotime value. Scale bar, 100 µm. **e**, Density plot displaying the computed pseudotime difference (*y* axis) versus the measured spatial distance (*x* axis) between all beads of the same cell state. Each dot is a pairwise comparison. The dotted line delineates cells with low spatial distance and large transcriptional divergence in the neural tube. **f**, Spatial plot showing the beads (top) within the dotted line (Fig. 2e) and the distribution of pseudotime values in an E9.5 embryonic trunk (array E9.5_3) that reflects neural tube patterning. Each dot represents a bead. Scale bars, 100 µm.

**Extended Data Fig. 8:**
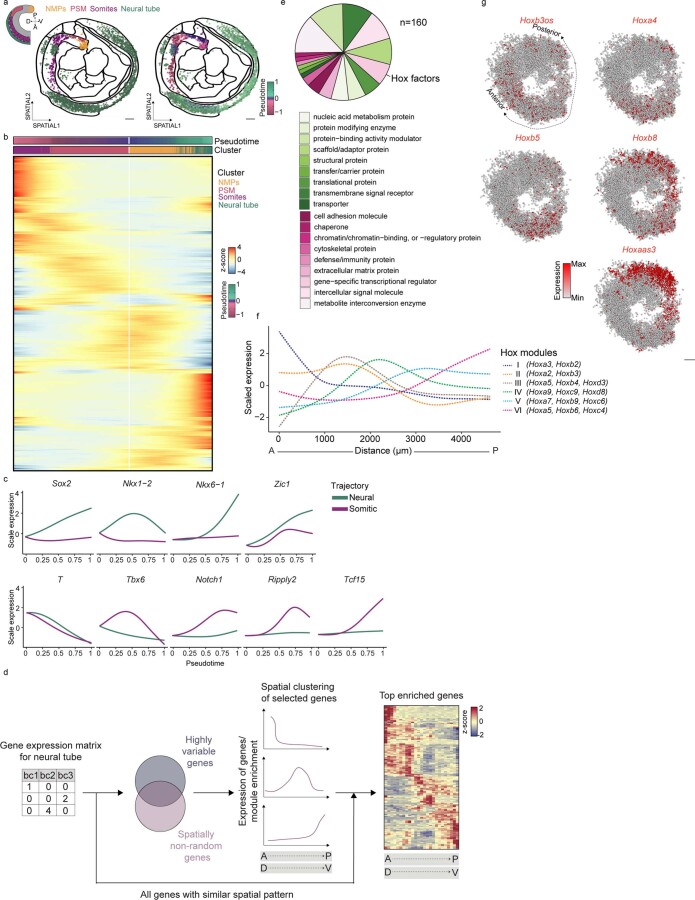
Transcriptional dynamics in the trunk region. **a**. Spatial plot showing beads from E9.5 embryo (array E9.5_6) corresponding to the NMPs, PSM, somites and neural tube clusters (left panel) and the corresponding pseudotime values (right panel). Each dot corresponds to a bead. Cell states are highlighted in the indicated colors. Scale bar, 200 µm. **b**. Heatmap showing the expression of pseudotime determining genes along the somitic and neural trajectories. Genes used for the heatmap are listed in Supplementary Table 6. **c**. Line plot showing gene expression of selected genes along the neural and somitic trajectories. *y-*axis represents scaled gene expression. **d**. Schematic workflow of the ‘axis profiling’ tool. **e**. Pie chart displaying differentially expressed genes along the anteroposterior axis divided by cellular and molecular function. Genes are listed in Supplementary Table 7. **f.** Line plot showing the spatial distribution along the anterior-posterior axis of the Hox modules’ expression (I-VI). **g**. vISH for *Hox* genes in 3D virtual E9.0 stage embryo. Scale bar, 200 µm. D, dorsal; V, ventral; A, anterior; P, posterior.

While the anterior neural tube develops into the brain, the posterior portion generates the future spinal cord during trunk elongation. The embryonic trunk consists of morphologically diverse structures with distinct developmental origins that ensure correct axial elongation and body plan segmentation (Fig. 3a)^58–60^. As the embryo develops, axial progenitor cells acquire a bipotent differentiation potential that allows the generation of neuroectodermal and mesodermal derivatives^58–60^. Specifically, these cells, known as neuromesodermal progenitors (NMPs), generate the posterior portion of the neural tube and the paraxial mesoderm via the determination front (pre-somitic (PSM) and somitic mesoderm) (Fig. 3a)^58–60^. To profile gene expression regionalization in the developing trunk in 3D, we first mapped the cell states corresponding to the NMPs, PSM and somites (somitogenesis trajectory) onto our E8.5 virtual embryo (Fig. 3b). The vISH of *Tbx6*, *Ripply2* and *Meox1* further confirmed the organized gene expression patterns along the AP and right-left symmetry involved in somitogenesis (Fig. 3b). Next, to understand how a regionalized gene expression signature impacts developmental dynamics, we profiled trunk developmental trajectories by combining transcriptional pseudotime measurements with spatial information. UMAP and spatial visualization revealed a continuum of transcriptomic states along the somitic and neural trajectories (Fig. 3c,d and Extended Data Fig. 8a–c). We next investigated this relationship in more detail and calculated the transcriptional and spatial distances for every bead to every other bead within each trunk tissue in an E9.5 embryo (‘pseudotime’ and ‘spatial’ distances, respectively) (Fig. 3e)^61–63^. Interestingly, we discovered that changes in transcriptomes are not necessarily proportional to cell–cell distances and that their relationship is tissue-specific. In particular, progenitor cells (NMPs and PSM) occupy a small region of the embryo, showing low spatial distance distributions concurrent with low transcriptional variability (Fig. 3e). On the other hand, more differentiated cells (neural tube and somites) are widely distributed along the trunk region (high distance distribution), even in cases of low transcriptional differences (Fig. 3e). We also observed a group of proximal cells in the neural tube characterized by notable transcriptional variability that, when mapped to spatial arrays, represented local DV patterning (Fig. 3e dotted line and Fig. 3f). Together, our findings show that during trunk development, progenitors differentiate into subsequent cell states in a restricted spatial domain before dispersal.

### Emerging patterns along the neural tube axes

**Fig. 4:**
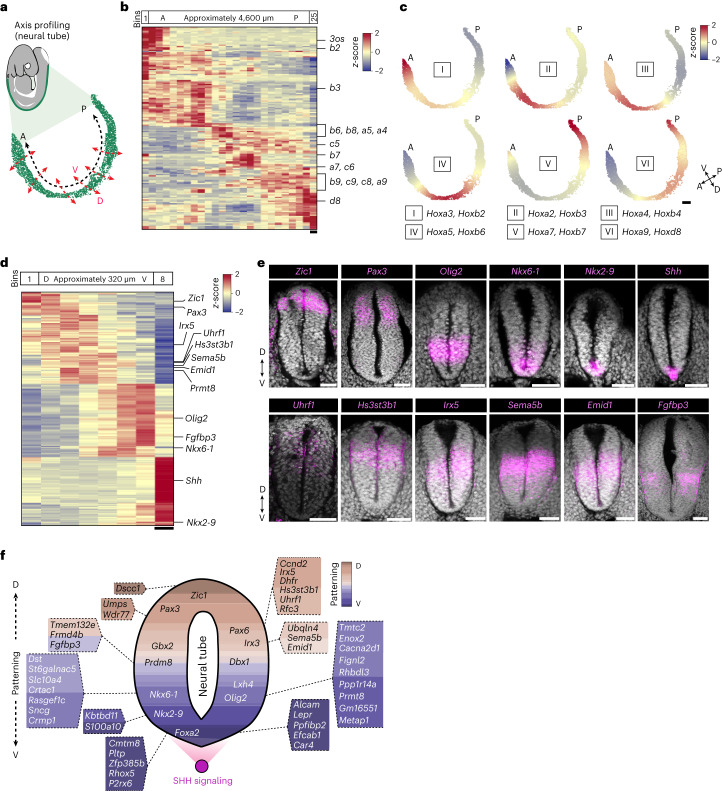
Neural tube profiling along the AP and DV axes. **a**, Schematic of an E9.5 stage embryo, with beads corresponding to the neural tube (green) and the denoted axes along which profiling was performed (array E9.5_10). **b**, Heatmap showing the top 160 genes with expression patterns (row *z*-score-normalized, filtered for a log fold change greater than 0.05 and a false discovery rate (FDR) < 0.01; see Supplementary Table 7 for the list of genes) in the 25 generated spatial bins along the AP axis. Expression of *Hox* genes is highlighted (right). Scale bar, 184 µm. **c**, Spatial plots showing the normalized expression (bin *z*-score) of *Hox* modules (I, II, III, IV, V, VI) in the E9.5 (array E9.5_10) neural tube. Two representative genes for each module are indicated. Scale bar, 100 µm. **d**, Heatmap showing the top 160 genes with expression patterns (row *z*-score-normalized, filtered for a log fold change greater than 0.05 and FDR < 0.01; see Supplementary Table 7 for the list of genes) in the eight generated spatial bins along the DV axis. Expression of known patterned and our identified genes are highlighted. Scale bar, 40 µm. **e**, RNA–FISH showing validation for known (top**)** and our identified (bottom) patterns along the DV axis of the neural tube. A single tissue slice obtained from the posterior portion of the neural tube is shown in the images. *n* = 3 embryos with reproducible staining pattern for each profiled target from WT embryos. Scale bars, 50 µm. **f**, Schematic showing the spatial patterns of known (within the tube) and our identified (outside the tube) genes along the DV axis of the E9.5 stage neural tube.

**Extended Data Fig. 9:**
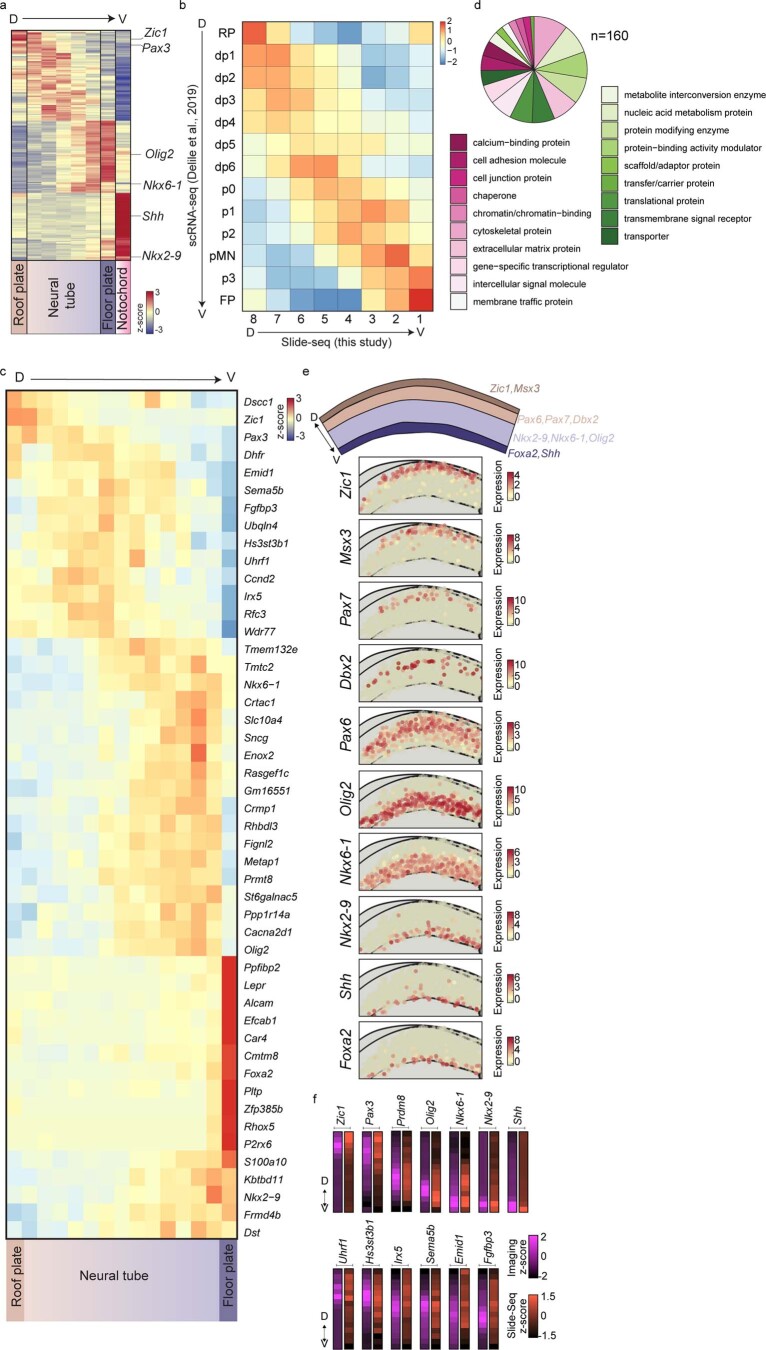
Neural tube dorsoventral patterning. **a**. Heatmap showing the top 160 genes with gene expression regionalization in one of the eight generated bins along the dorsoventral axis (genes filtered by FDR < 0.01 and logFC>0.05 then ranked by FDR). A selected set of previously known genes associated with dorsoventral patterning (black) are highlighted on the right. Specific structures along the axis are highlighted at the bottom of the heatmap. Genes are row z-score normalized and listed in Supplementary Table 7. **b**. Heatmap showing column scaled *z*-score of Pearson correlation coefficients comparing the Slide-seq dorsoventral bins and the identified clusters from a neural tube single-cell reference^26^. RP, roof plate; dp, dorsal progenitors; pMN, motor neuron progenitors; FP, floor plate. **c**. Heatmap showing an extended subset of the genes that display gene expression regionalization in one of the eight generated bins along the dorsoventral axis (genes filtered by FDR < 0.01 and logFC>0.05 then ranked by FDR). Specific structures along the axis are highlighted at the bottom of the heatmap. Genes are row z-score normalized and listed in Supplementary Table 7. **d**. Pie chart displaying differentially expressed genes along the dorsoventral axis divided by cellular and molecular function. Genes are listed in Supplementary Table 7. **e**. Schematic (top panel) and spatial gene expression plots of the known neural tube patterning genes in array E9.5_2. Each dot denotes a bead. The color scale depicts normalized gene expression. D, dorsal; V, ventral; A, anterior; P, posterior. **f**. RNA-FISH and Slide-seq quantifications for each profiled gene from Fig. 4e. Genes are bin *z*-score normalized (magenta: RNA-FISH; orange: Slide-seq). D, dorsal; V, ventral.

As the trunk develops and tissues extend in space, the transcriptional differences along their length determine the positional identity of various cellular states. For example, between E8.5 and E10.5, the neural tube folds from the neural plate and undergoes patterning to establish the cellular stratification for the future spinal cord^16,17,19–21,62–64^. Regionalized gene expression programs guarantee further diversification of neuronal types along the AP and DV axes^16,17,19–21,62–64^. To understand the genetic programs involved in establishing the discrete progenitor domains along the neural tube, we isolated the corresponding beads and searched for spatial co-expression patterns along the AP (approximately 4,600 µm) and DV (approximately 320 µm) axes (‘axis profiling’) (Fig. 4a and Extended Data Fig. 8d). We identified distinct expression patterns along the neural tube AP axis for genes involved in several cellular and molecular functions (Extended Data Fig. 8e). As expected, *Hox* genes were among the most highly localized genes within this axis (Fig. 4b)^65,66^. HOX factors interact with each other to regulate transcriptional programs. To identify putative functionally collinear groups, we performed *Hox* gene module analysis combined with spatial resolution and found six distinct modules of *Hox* gene expression, from the most anterior module (*Hox* module I, comprising *Hoxb2* and *Hoxa3*) to the most posterior one (*Hox* module VI, containing *Hoxd8* and *Hoxa9* (Fig. 4c and Extended Data Fig. 8f,g). Next, we examined the DV axis of the neural tube and were able to resolve and annotate well-studied structures like the notochord, the floor plate, the actual neural tube and the roof plate based on their spatially restricted transcriptional signature (Fig. 4a,d and Extended Data Fig. 9a,b)^29^. Among the most spatially constrained genes, we found well-known lineage-defining markers like *Zic1*, *Pax3*, *Olig2*, *Nkx6-1* and *Nkx2-9*, which we further confirmed using RNA–fluorescence in situ hybridization (FISH) (Fig. 4d–f and Extended Data Fig. 9c–e). We also identified 43 additional genes in the early mouse neural tube at the E9.5 stage that appear to exhibit a patterned expression along the DV axis in the ventricular zone containing progenitors (Fig. 4d–f, Extended Data Fig. 9f and Supplementary Table 7). Our results show the utility of the axis profiling tool in detecting well-studied gene expression patterns along the neural tube axis and its application in the de novo discovery of genes with locally restricted expression.

### Conflicting identity of ectopic neural tubes in *Tbx6* mutants

**Fig. 5:**
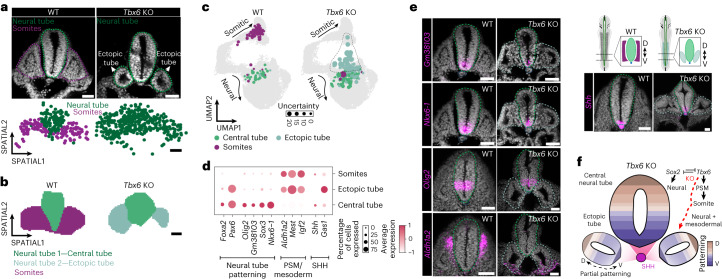
Slide-seq profiling of *Tbx6* KO embryos. **a**, Transverse section of DAPI stained embryos (top) and spatial plot (bottom) showing the annotated tissue morphologies (somites, neural tube) and corresponding cell states in E9.5 WT and KO embryo. The KO experiment was independently performed five times in total with *n* > 5 embryos per experiment, and consistently yielded the same phenotype. The Slide-seq experiment was performed on one representative transversal section for WT and two for KO embryos. **b**, Spatial grid map showing the organization of neural tube 1, 2 and somitic cells in WT and *Tbx6* KO embryos. **c**, UMAP showing the projection of cells assigned to the indicated clusters on the trunk trajectory (Fig. 3c), with the size of the dots representing the degree of uncertainty of mapping to the respective position. **d**, Dot plot showing the expression of the indicated genes in the three clusters (dot size is percentage of cells per cluster; color is cluster average normalized expression). **e**, RNA–FISH of the indicated genes in a transversal section of an E9.5 WT and KO embryos. The dotted lines in the schematic denote the AP position within the trunk from which sections were obtained. A representative section from WT and KO embryos at E9.5 is shown. The expression pattern was verified in two of the KO experiments with *n* > 3 embryos per experiment. Scale bars, 50 µm. **f**, Schematic showing the transcriptional identity and patterning characteristic of the ectopic neural tubes that arise in the absence of *Tbx6* expression, highlighting their conflicting transcriptional identity.

**Extended Data Fig. 10:**
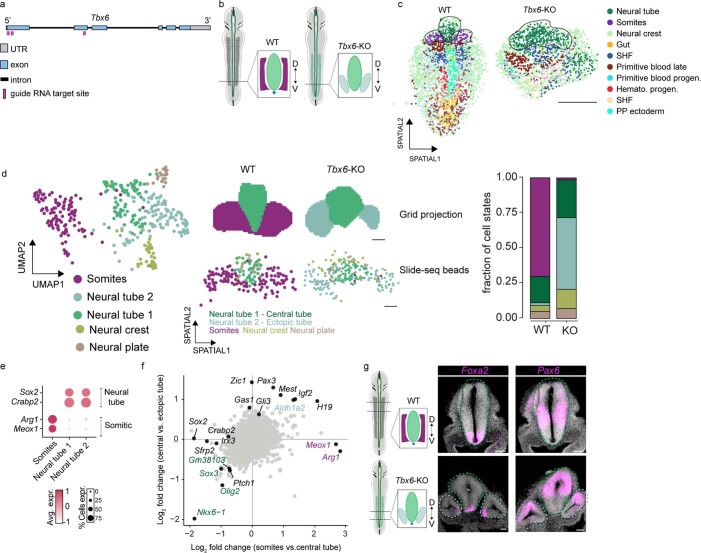
*Tbx6*-KO spatial profiling. **a**. Schematic of *Tbx6* gene ablation strategy. Guide RNAs target the denoted exons. UTR, untranslated region. **b**. Schematic showing the plane of cryosectioning for Slide-seq experiment. **c**. Spatial plot of all cell states in the complete WT and *Tbx6*-KO trunk transversal arrays. Dotted lines denote the region that was used for further analysis. Each dot corresponds to a bead. Each color represents an individual state. Scale bar, 200 µm. **d**. UMAP, spatial and fraction plot showing the filtered and *de novo* annotated cell states. **e**. Dot plot depicting the expression of neural tube and somitic marker genes in the somites, neural tube 1 and 2 clusters. The size of the dots represents the % of cells expressing the gene, and the color represents the cluster average normalized expression level. **f**. Scatter plot showing differentially expressed genes between somitic vs central tubes clusters and central vs ectopic tubes clusters (genes filtered by FDR < 0.01. Complete list of genes in Supplementary Table 9. **g**. RNA-FISH of *Foxa2* and *Pax6* in a transversal section of the neural tube in WT and *Tbx6*-KO embryos. The schematic represents the anteroposterior position where the transversal sections were profiled. Scale bar, 50 µm. D, dorsal; V, ventral; WT, wild type; KO, knockout.

After cataloging the spatial transcriptome underlying the developing neural tube axes, we investigated a classic embryonic mutant phenotype where ectopic neural tubes arise. The T-box transcription factor TBX6 is expressed in the PSM and is required for somite segmentation and specification^10^. In embryos lacking *Tbx6*, ectopic neural tubes arise at the expense of the somitic compartments (Fig. 5a)^11,67^. To assess the precise molecular identity of the ectopic neural tubes, we used CAS9-based disruption of *Tbx6* in zygotes and performed Slide-seq on E9.5 wild-type (WT) and *Tbx6* mutant (*Tbx6* knockout (KO)) transversal embryo sections, focusing on the posterior segment of the trunk region where multiple tubes arise in the absence of *Tbx6* (Fig. 5a and Extended Data Fig. 10a–c)^26,68–70^. As expected, we observed an overrepresentation of beads assigned to the neural tube cluster in *Tbx6* KO embryos compared to WT controls, along with a commensurate lack of somitic cells (Fig. 5a).

Next, we reclustered the beads having a neural and somitic identity to more closely inspect differences between the neural tubes in the *Tbx6* KO embryos. We found that neural tube cells are subsequently resolved into four transcriptional subclusters, which we labeled as neural crest, neural plate and two main neural tube clusters (neural tubes 1 and 2, Extended Data Fig. 10d)^71–75^. Interestingly, when we spatially assigned the two neural tube cluster cells on WT and *Tbx6* KO arrays, we discovered that neural tube 1 cells mapped to the central tube in both genotypes. In contrast, neural tube 2 cells mapped to both sides of the central tube exclusively in the *Tbx6* KO, suggesting that ectopic tubes are characterized by a distinct transcriptional state (Fig. 5b). Specifically, cells of ectopic tubes display a transcriptional identity that is in between the somitic and neural cells, despite their predicted neural identity (Fig. 5c and Extended Data Fig. 10e,f). We observed high levels of mesodermal-specific genes including *Aldh1a2* and *Mest* (Fig. 5d,e). Concomitant with the expression of mesodermal markers, ectopic tube-assigned cells had reduced or absent expression of classic neural tube patterning marker genes such as *Olig2*, *Gm38103*, *Sox3*, *Nkx6-1* and *Shh* (Fig. 5d,e). Nonetheless, the mesodermal signature was overlayed with the conflicting expression of other characteristic neural tube patterning genes, such as *Foxa2* and *Pax6* (Extended Data Fig. 10g)^11^.

In summary, our spatial transcriptomic analysis of the ectopic tubes in *Tbx6* mutant embryos identified cells that acquire a mixed transcriptomic identity, characterized by the expression of mesodermal genes and partial DV gene expression patterning (Fig. 5f).

## Discussion

We performed embryo-wide spatial transcriptomic profiling using Slide-seq to decipher the tightly regulated gene expression patterns of approximately 27,000 genes in developing embryos at the onset of organogenesis. The reconstruction of digital 3D embryos using sc3D enabled the quantitative exploration of gene expression patterns and gradients on a virtual in situ basis. Combined with the development of sc3D-viewer (a napari plugin)^76^, an accessible, interactive and user-friendly visualization platform to register and explore 3D spatial genomic data, we facilitate the rapid and seamless exploration of cell type distribution and gene expression patterns along any given developmental axis, including the possibility to reconstruct tissues from other spatial transcriptomic datasets.

Spatially resolved single-cell sequencing methods continue to evolve rapidly^37,38,40,77–79^. The increase of available datasets will require faster and more precise computational approaches to take full advantage of the added spatial information. sc3D contributes toward the in-depth analysis of the topology and geometry of gene expression and co-expression patterns, providing the infrastructure to start modeling gene expression diffusion, and their interaction in embryonic tissues. The sc3D data structure has been purposely designed to be close to the one of imaging-based cell tracking algorithm outputs. This similarity will ease the porting of cell tracking-based inter-sample alignment algorithms, such as Tardis, to the spatial transcriptomic field^31^.

The dynamics at which transcriptomes evolve as progenitors differentiate in their spatial distribution has been challenging to explore. The analysis of differentiation trajectories in the embryonic brain and trunk regions revealed several discrete domains in which transcriptional changes converge or diverge spatially, indicating a non-linear and tissue-specific relationship. We observed that spatially dependent relationships were frequently associated with regions characterized by high intercellular signaling gradients. These changes may reflect the specification of sublineages, progenitor pool migration, differentiated subtypes maturation or regionalization of specific fates. Additional studies combining lineage recording, spatial information and single-cell transcriptomic profiling might help resolve the causal relationship between gene expression programs, spatial allocation and cell fate specification.

The transcriptional rewiring along the AP and DV axes of the developing neural tube defines discrete gene expression domains instrumental in controlling future cellular diversification^16,17,19–21,62–64^. We discovered several interesting genes that display regionalized gene expression patterns along these axes, including epigenetic and metabolic regulators that require further investigation to determine their molecular role and functional implications.

As an example for leveraging spatial information in a perturbation experiment, we provided a detailed transcriptomic characterization of the molecular identity of the ectopic neural tubes that arise in *Tbx6* mutant embryos. Unexpectedly, what has been historically assigned as additional neural tubes are morphologically tubular structures with incomplete patterning and continued expression of mesodermal genes that are usually associated with non-epithelial and mesenchymal cell identities. This suggests a decoupling of transcriptional programs and morphogenetic outcomes during embryonic development, with signaling gradients, extracellular matrix and mechanical clues probably playing crucial roles^80^. While *Tbx6* mutant embryos exhibit a distinct and well-characterized phenotype, many other genes may cause less obvious morphological changes and hence will benefit from a similar spatiotemporal characterization to define their developmental roles.

Our accessible resource of spatial transcriptomic maps at the onset of organogenesis and supporting computational tools will help the continued exploration of mammalian development. Furthermore, the framework presented in this study could be implemented to conduct molecular spatial phenotyping on many additional perturbations. Lastly, by combining lineage mapping and multi-omic analysis, a comprehensive map of the gene regulatory network during embryogenesis could be developed based on this work.

## Methods

All experiments described in this article comply with the relevant ethical regulations at the respective institutions. All experiments were approved by the Landesamt für Gesundheit und Soziales. All animal procedures were performed according to animal welfare guidelines and regulations approved by the Max Planck Institute for Molecular Genetics (G0243/18-SGr1_G and ZH120).

### Animal work and embryo preparation (WT and KO embryos)

WT E8.5, E9.0 and E9.5 embryos were dissected from the uteri of naturally mated CD-1 mice in 1× HBSS (catalog no. 14175053, Gibco) on ice. Embryos were staged based on morphology, size and somite number (3–5 somite pair stage for E8.5, 10–12 somite pair stage for E9.0 and 15–18 somite pair stage for E9.5). Extra-embryonic tissues were removed from E9.5 embryos before processing. Embryos were washed in cold 1× HBSS with 2 U ml^−1^ RNase inhibitor (catalog no. N8080119, Thermo Fisher Scientific) and embedded in O.C.T. solution (catalog nos. 23-730-571 and 23-730-572, Thermo Fisher Scientific). Embryos in O.C.T. were oriented under a stereoscope, immediately placed on dry ice for flash-freezing and ultimately stored at −80 °C. *Tbx6* mutant embryos were generated by a previously established protocol^26,68,69,70^. Briefly, in vitro fertilized (IVF) zygotes were electroporated with Alt-R CRISPR–Cas9 RNP complex with guides targeting three different exons of *Tbx6* (Supplementary Table 10). Embryos that developed to blastocyst stage were retransferred to CD-1 pseudo-pregnant surrogate animals as described. All *Tbx6* mutant embryos isolated at E9.5 showed the mutant phenotype (enlarged tail bud, ectopic neural tubes). The trunk region was dissected from WT and mutant embryos (removed parts above the limb bud and heart), embedded in O.C.T. solution and frozen at −80 °C.

Animals were kept under specific pathogen-free conditions in individually ventilated cages at 22 ± 2 °C, 55 ± 10% humidity with a 12-h light–dark cycle (6:00–18:00). IVF was performed with B6D2F1 oocyte donors (aged 7–9 weeks; Envigo) and sperm was isolated from B6.CAST F1 males (aged 2 months, generated in-house by breeding C57BL/6J females and CAST/EiJ males). For the embryo transfer experiments, pseudopregnant CD-1 female mice (Hsd:ICR; 9–12 weeks old; 21–25 g; Envigo) were mated with vasectomized males (Swiss Webster; older than 13 weeks; Envigo).

### Cryosectioning for Slide-seq V2

Fresh-frozen O.C.T. blocks with mouse embryos were equilibrated to −20 °C in a cryostat (CM1950, Leica Biosystems), mounted onto a cutting block with O.C.T., sliced at a 10-μm thickness and then overlaid and melted onto sequenced spatial arrays^33,39^. Sagittal sections for whole embryos were collected at the following intervals: E8.5 embryos, 30-μm distance; E9.0 embryos, 20-μm distance; and for E9.5, sections from mid-volume were collected from three independent embryos. One for the head region and another for the thoracic and trunk region from each embryo (Fig. 1a and Supplementary Table 1). An E9.5 transversal section of the trunk region (posterior trunk corresponding to the somite-neural tube region) was collected for WT and *Tbx6* mutant embryo with a 10-µm section thickness.

### Whole-embryo RNA–FISH

Whole-embryo RNA–FISH was performed according to the protocol from Molecular Instruments with some modifications. Briefly, embryos fixed overnight with 4% paraformaldehyde (PFA) at 4 °C were washed three times for 10 min each with 1× PBS with 0.1% Tween 20 (PBST) at 4 °C. Embryos were dehydrated in an increasing concentration series of methanol + PBST washes, for 10 min each wash at 4 °C (25% methanol; 50% methanol; 75% methanol; 100% methanol). Embryos were stored at −20 °C overnight or longer. Next, embryos were rehydrated in a decreasing concentration series of methanol + PBST washes, for 10 min each wash at 4 °C (100% methanol; 75% methanol; 50% methanol; 25% methanol; 100% PBST). After two washes for 10 min at 4 °C in PBST, embryos were bleached with 6% hydrogen peroxide (for endogenous peroxidase activity in blood cells) at 4 °C for 20 min. After two washes in PBST for 10 min each at 4 °C, embryos were treated with 10 µg ml^−1^ proteinase K (catalog no. EO0491, Thermo Fisher Scientific) for the indicated time at room temperature (E9.5: 10 min). After two washes with PBST for 15 min each, embryos were postfixed in 4% PFA for 15 min at room temperature and washed three times in PBST for 15 min each step. Embryos were then prepared for hybridization by incubating in hybridization buffer at 37 °C for 1 h. Probes were resuspended in hybridization buffer at a concentration of 1 pM and incubated with embryos overnight at 37 °C. Embryos were washed four times with probe wash buffer for 15 min each wash at 37 °C, followed by three washes in 5× SSCT hybridization buffer + 0.1% Tween 20. Fluorescent hairpins were prepared as described by the manufacturer at a concentration of 0.06 µM each hairpin in amplification buffer. Embryos were then incubated in amplification buffer before incubation with hairpin probes overnight at room temperature in the dark. Excess probes were removed by five washes of 15 min each step in 5× SSCT at room temperature in the dark. Nuclei were counterstained by incubation with 2 µg ml^−1^ 4,6-diamidino-2-phenylindole. The buffers and probe sequences used in this study are available at Molecular Instruments and their unique ID can be found in Supplementary Table 10. After hybridization, embryos were embedded in O.C.T. and frozen at −80 °C. O.C.T. blocks were sectioned to obtain transversal sections of the trunk region and neural tube at 30-µm thickness. Embryos and sections were imaged with a ZEISS LSM-880 confocal microscope at 10×, 20× magnification, averaging four times per frame and 10-µm z-stacks. Images were processed with the ImageJ software. The Plot Profile function was used to perform the signal intensity along a user-defined axis for each fluorescent channel.

### Slide-seq V2

The Slide-seq V2 protocol was used to generate all the sequencing libraries. Bead synthesis, array sequencing, image processing and base calling were performed^33,39^ as described below. Briefly, the 10-μm barcoded beads were synthesized in-house by ChemGenes with a 14-bp spatial barcode separated by a 14-bp linker sequence, an 8-bp unique molecular identifier (UMI) sequence and a 20-bp poly(T) tail. The bead arrays were prepared by resuspending the synthesized beads in 10% dimethylsulfoxide at a concentration of 20,000–50,000 beads per microliter. Then, 10 μl of the bead solution was pipetted into each position on the gasket. The coverslip-gasket filled with bead solution was centrifuged at 750*g* for at least 30 min at 40 °C until the surface was dry. To extract the spatial barcodes, arrays were sequenced using Bioptechs FCS2 flow cells with an RP-1 peristaltic pump (Rainin) and a modular valve positioner (Hamilton MVP). During sequencing, flow rates between 1 and 3 ml min^−1^ were used. Imaging was obtained with Nikon Plan Apo 10×/0.45 objective. Sequencing was performed using a sequencing-by-ligation approach. Base calling from the images was performed using the custom MATLAB package PuckCaller (https://github.com/MacoskoLab/PuckCaller).

### Slide-seq V2 library generation

The complete protocol for the Slide-seq V2 library preparation can be found at https://www.protocols.io/view/library-generation-using-slide-seqv2-81wgb7631vpk/v1?version_warning=no. Briefly, arrays covered with freshly cut tissue sections were transferred to tubes containing 6× SSC supplemented with RNAase inhibitor (1:20 concentration, catalog no. 30281-2, NxGen, Lucigen) and incubated for 15 min at room temperature. Arrays were then dipped in 1× reverse transcriptase buffer and then transferred to tubes containing the reverse transcription mix (Maxima 1× reverse transcriptase buffer, 1 mM deoxynucleoside triphosphates (dNTPs), 2 U ml^−1^ RNase inhibitor, 2.5 mM template switch oligonucleotides (catalog no. 339414YCO0076714, QIAGEN) and 10 U ml^−1^ Maxima H minus reverse transcriptase) for 30 min at room temperature followed by a 90-min incubation at 52 °C. Proteinase K (1:50 concentration) and tissue clearing solution were added to the same tube and incubated at 37 °C for 30 min. Beads were then removed from the glass slide by pipetting up and down a few times and resuspended in TE-TW solution (10 mM Tris, pH 8.0, 1 mM EDTA, 0.01% Tween 20) subjected to two TE-TW washes followed by 2-min centrifugation at 3000*g*. After removing the supernatant, beads were resuspended in 200 μl of exonuclease I mix (20 μl of 10× ExoI buffer, 10 μl of ExoI, New England Biolabs) and incubated at 37 °C for 50 min. Beads were then washed twice in TE-TW, followed by a 5 min incubation in 0.1 N NaOH at room temperature and another TE-TW wash. Second-strand synthesis was performed on beads by adding 200 μl second-strand mix (Maxima 1× reverse transcription buffer, 1 mM dNTPs, 10 mm dN-SMRT oligonucleotides, 0.125 U ml^−1^ Klenow fragment) and incubating at 37 °C for 60 min. Next, beads were washed three times in TE-TW before amplification with whole transcriptome amplification PCR (1× Terra Direct PCR mix buffer, 0.25 U ml^−1^ Terra polymerase, 2 mM TruSeq PCR handle primer and 2 mM SMART PCR primer) with the following conditions: 95 °C 3 min; 4 cycles of 98 °C for 20 s, 65 °C for 45 s, 72 °C for 3 min and 9 cycles of 98 °C for 20 s, 67 °C for 20 s, 72 °C for 3 min and 72 °C for 5 min. The PCR product was cleaned up by 0.6× solid-phase reversible immobilization twice and resuspended to a final volume of 10 μl. Then, 1 μl of the library was quantified on either an Agilent Bioanalyzer High Sensitivity DNA chip or Agilent TapeStation High Sensitivity D500 DNA screenTape. Then, 600 pg of the PCR product was used as input to generate Illumina sequencing libraries by tagmentation with an Illumina Nextera XT kit (catalog no. FC-131-1096). The library was amplified with TruSeq 5 and N700 series barcoded index with the following conditions: 72 °C for 3 min; 95 °C for 30 s; 12 cycles of 95 °C for 10 s, 55 °C for 30 s, 72 °C for 30 s and 72 °C for 5 min. After cleaning up, final libraries were sequenced on a NovaSeq S2 or S4 flowcells with approximately 300 million reads per array for E8.5_Embryo_1 and E9.5 embryos, approximately 200 million reads per array for E8.5_Embryo_2 and E9.0 and approximately 50 million reads per array for WT and *Tbx6* KO transversal sections.

### Slide-seq data processing and cell state annotation

The sequenced reads were processed using the Slide-seq tools pipeline (https://github.com/MacoskoLab/slideseq-tools) to generate the gene count matrix and match the bead barcode between array and sequenced reads. Most of the downstream analysis was performed in R (v.4.1.0), except the RNA velocity analysis performed in Python (v.3.8.3, v.3.9.0, v.3.10). The gene count matrix and bead spatial coordinates were processed using Seurat (v.3.0.0, v.4.0.2)^81^. Beads with more than 200 counts and less than 20% mitochondrial gene counts were retained for further analysis. The data from each stage were merged due to minimal batch effect and analyzed together. For the E8.5 replicate and E9.5 data, the top 3,000 highly variable genes were used in FindVariableFeatures and the top 40 principal components from the principal component analysis (PCA) (RunPCA from Seurat). Seurat label transfer was used to obtain cell state annotation, with the functions FindTransferAnchors and TransferData. For the second E8.5 replicate and E9.0 data, we used robust cell type decomposition (RCTD)^82^ to annotate the cell state because it is more robust to lower UMI counts. Data from the E8.5 stage in our previous study were used as reference data for the Seurat label transfer function and the RCTD function^26^. Plots were generated with ggplot2 (v.3.1.0).

### Reclustering of cell types in the brain

To obtain a better understanding of the different cell types in the brain of E9.5 embryos, we performed de novo clustering from the beads corresponding to the E9.5 stage arrays, further annotating and refining the identities using the Seurat pipeline (resolution = 1), and manually annotated each of the 30 clusters using known marker genes and label transfer results.

### Differential expression analysis on brain boundaries

First, we combined marker gene spatial velocities to identify genes expressed within the brain boundaries, *Fgf8* for the mid–hindbrain boundary, *Foxg1*, *Barhl2* and *Wnt8b* for the telencephalon–diencephalon boundary, and *Barhl2* and *Pax6* for the diencephalon–midbrain boundary. Next, we calculated the distance between each bead to the boundaries and selected beads within a 300-µm distance. Genes whose expression correlated with the distance to the brain boundaries were identified using an edgeR (v.3.34.1) quasi-likelihood model (glmQLFit function)^83^. The top 40 genes ranked according to FDR were selected for heatmap visualization (Complex heatmap, v.1.99.5) in Fig. 2c.

### Identification of spatially variable genes in the developing eye

The beads associated with developing eye were identified in a semi-supervised way. First, the genes correlated with the known marker genes *Rax*, *Vax1* and *Six6* were extracted using gene co-expression analysis. These genes were used as input for the PCA and clustering pipeline in Seurat; the top 5 principal components were used. Next, we used dbscan to spatially refine the clustering results and remove a few outliers in the eye cluster. Then, we compared the eye cluster to the forebrain cluster using the FindMarkers function and searched for new marker genes specifically expressed in the developing eye regions.

### 3D reconstruction and identification of spatially variable genes in 3D

The sc3D reconstruction and associated analysis is described in detail in the Supplementary Information and can also be found at https://github.com/GuignardLab/sc3D.

### RNA velocity analysis on Slide-seq data

We adapted the scVelo (v.0.2.4) package^84^ to analyze the RNA velocity at the spatial axis. Using the tutorial at https://scvelo.readthedocs.io/, exonic and intronic counts from each bead were extracted from the data and used as input. The stochastic model with default parameters was used to compute the velocity of each bead. Next, the velocity vectors were projected to the physical space. For visualization, the velocity vectors were computed according to a 50 × 50 μm grid; the 50 nearest neighbors were selected in each grid to calculate the average velocity. The length of velocity with regard to the speed of the transcriptomic changes was calculated by taking the average length of the velocity vector from the neighbors (*n* = 50) of a bead. The velocity confidence represents the coherence of the velocity direction. It was calculated by taking the sum of cosine angle between the velocity vector and its neighbors (*n* = 50). The function rank_velocity_genes was used to identify and rank genes that contribute to the vector field, which means that genes are actively transcribed and have more nascent mRNA as cells differentiate.

### Trajectory analysis in the trunk region

Beads annotated as NMPs, somites and neural tube from E8.5 and E9.5 were selected. Next, beads with a prediction score lower than 0.6 were discarded to remove cell mixtures. The remaining beads from E8.5 and E9.5 were integrated using Harmony (v.0.1.0)^85^ with default parameters; then, UMAP dimensionality reduction (runUMAP) was performed based on an integrated matrix. Next, we used Monocle3 (v.1.0.0) to calculate the pseudotime from the UMAP output, according to the tutorial and using default parameters (https://cole-trapnell-lab.github.io/monocle3/docs/trajectories/). A generalized linear model with quasi-likelihood dispersion estimators from edgeR (v.3.34.1)^83^ was used to find the genes that correlated with the pseudotime trajectory. Briefly, as with the tutorial^86^ instructions, we used estimateDisp to estimate the gene-wise negative binomial dispersions, followed by glmQLFit or glmQLFTest to test genes that were significantly correlated with the pseudotime value, which was used as a covariate in the design matrix. Genes with an FDR < 0.01 and a log fold change greater than 0.05 were selected and plotted into a heatmap.

### Analysis of the relationship between transcriptional dynamics and spatial distance

After trajectory analysis in the trunk region, the pairwise distance between beads within each cell state were calculated based on their spatial distance and pseudotime difference. This analysis results in the generation of a spatial and pseudotime distance matrices. Next, the spatial distance and pseudotime distance of each pair were compared and plotted.

### Identification of spatial differentially expressed genes in the neural tube

The beads assigned to have a neural tube identity in array E9.5_10 were used for this analysis. Using Slingshot::slingshot (v.2.0.0)^87^, we calculated the principal curves from the beads’ spatial location and ordered the cells along the principal curves as their anterior to posterior distance. The distance of each bead to the convex hull of the neural tube was computed as the dorsal to ventral distance and split into eight equally spaced bins. We used SPARK (v.1.1.1)^88^ with default parameters to find spatially variable genes. We took the intersection of spatially variable genes from SPARK and the variable genes from Seurat as the input for the spatial module analysis. Dynamic time warping was applied to find genes with coherent spatial patterns that we defined as modules (in the Hox genes analysis). The Dtwclust (v.5.5.6) (https://CRAN.R-project.org/package=dtwclust) package was used for the analysis with the tsclust function (*k* = 8). The centroid of each module was calculated as their spatial pattern. Next, we used the same edgeR pipeline as the trajectory analysis to find more genes correlated with each spatial pattern. We selected genes with the same threshold and visualized them into a heatmap. The *Hox* genes were grouped as described in the previous section and the average expression of each module was smoothed using the gam::gam (v.1.2.0) function for visualization. To determine the protein classes that are enriched in the differentially and spatially variable genes, we used PANTHER (v.17.0)^89^.

### Analysis of *Tbx6* mutant Slide-seq data

The data from the transversal sections of WT and *Tbx6* mutant embryos were merged and analyzed due to the low batch effect. The neural tube and somite regions were extracted based on marker gene expression, cell type labels and tissue morphology. Because of the small sample size, the extracted beads were processed using the Seurat pipeline with different parameters. The parameters used were the following: select 1,000 genes using the FindVariableFeatures function; use the first 20 principal components in the FindNeighbors function; and consider 15 neighbors in the *k*-nearest neighbor calculation, using of a resolution of 0.8 in the FindClusters function and setting n.neighbors = 20, min.dist = 0.3 in the RunUMAP function. After de novo clustering, we annotated each cluster by its marker genes and excluded cluster 0 because it mainly contained low-quality beads. Clusters 4 and 5 corresponded to the neural crest and neural plate, respectively, ruling out further analysis. We computed the raster spatial density of each cluster using MASS::kde2d (v.7.3–54) and combined them by taking the highest density for each position, which is plotted in Fig. 5b. We mapped the WT and *Tbx6* mutant data to the E9.5 trunk dataset as a reference by calculating their mutual nearest neighbor using the fast mutual nearest neighbors correction^90^ method. For each bead in the *Tbx6* mutant data, we selected its ten nearest neighbors in the integrated PCA space as anchors. We computed the variance of the dimensionality reduced matrix from neighbors as a metric of projection uncertainty. The averaged UMAP coordinates of ten nearest neighbors for each bead were projected on the reference UMAP, with size representing the projection uncertainty. The FindAllMarkers function was used to find the marker genes for each cluster. We then applied the FindMarkers function to compare two clusters (somite versus central neural tube and central neural tube versus ectopic neural tube), with the fold change of each gene.

### Statistics and reproducibility

All attempts at replicating the observations were successful (indicated below). Preselection of samples was performed, if indicated (below). No samples or data were excluded from the analysis, unless otherwise stated in the Methods. All comparisons (*Tbx6* KO) were performed with control samples from the same experiment. Sequencing and downstream processing and analysis were independent of human intervention. Blinding was not relevant because this was not an intervention study; pipelines were executed uniformly across all samples, allowing unbiased analysis. No statistical methods were used to predetermine sample sizes, but our samples are similar to those reported in previous publications^29–39^.

A two-sided Wilcoxon rank-sum test was used to identify marker genes and Bonferroni correction was used for the multiple comparisons in Fig. 2c, Extended Data Figs. 6d and 10f, Supplementary Tables 5 and 6, and Supplementary Tables 8 and 9. All other tests are described in the figure legends. The FDR and *P* values used (FDR < 0.01 and log fold change greater than 0.05) are indicated in the legends.

In Extended Data Fig. 4c, elements in the box plots are as follows: middle line, median; box plot limits, upper and lower quartiles; whiskers, s.d.

The Slide-seq experiments involving WT embryos were performed on two whole E8.5, one E9.0 and 13 partial sections from three embryos at the E9.5 stage. Embryos were obtained from at least three independent isolation experiments and staged for somite count (3–5 somite pair stage for E8.5; 10–12 somite pair stage for E9.0; 15–18 somite pair stage for E9.5), which are the representative embryos shown in Extended Data Fig. 1a. The Slide-seq experiment involving WT and *Tbx6* KO embryos was performed on one (WT) and two (*Tbx6* KO) transversal sections. The *Tbx6* KO experiment was performed independently five times (in total) to verify the phenotype, which was reproduced in every embryo across all experiments. Sections were obtained from the posterior part of the trunk (the representative image of the section collected before Slide-seq is shown in Fig. 5a). Whole-mount in situ hybridization was performed once in WT E9.5 stage embryos for the indicated number (*n* = 3) and showed reproducible results (Extended Data Fig. 2b). The RNA–FISH experiments were performed from two of the independent experiments with the indicated number (*n* = 3 embryos) and showed reproducible results, with one representative image shown in Extended Data Figs. 5c, 7f, 9f and 10g, and Figs. 4e and 5e.

### Reporting summary

Further information on research design is available in the Nature Portfolio Reporting Summary linked to this article.

## Online content

Any methods, additional references, Nature Portfolio reporting summaries, source data, extended data, supplementary information, acknowledgements, peer review information; details of author contributions and competing interests; and statements of data and code availability are available at 10.1038/s41588-023-01435-6.

## Supplementary information

Supplementary Information Supplementary Figs. 1 and 2, Methods and Notes on the available data visualization.

Reporting Summary

Peer Review File

Supplementary Tables Supplementary Tables 1–10.

Supplementary Movie 1 3D view of E8.5_Embryo1.

Supplementary Movie 2 3D view of E8.5_Embryo2.

Supplementary Movie 3 3D view of E9.0.

Supplementary Movie 4 vISH and whole-mount in situ hybridization of localized genes (heart and brain) in E8.5_Embryo2.

Supplementary Movie 5 3D vISH of localized genes in the heart in E8.5 embryo_1.

## Data Availability

Raw and processed data can be downloaded from the Gene Expression Omnibus under accession no. GSE197353. The input object for 3D visualization for the following embryos can be downloaded: E8.5_Embryo1 (https://figshare.com/s/1c29d867bc8b90d754d2); E8.5_Embryo2 (https://figshare.com/articles/dataset/E8_5_Embryo2_h5ad/21695849/1); E9.0 (https://figshare.com/articles/dataset/E9_0_Embryo_h5ad/21695879/1). Individual Slide-seq arrays can be visualized at https://cellxgene.cziscience.com/collections/d74b6979-efba-47cd-990a-9d80ccf29055. Whole-mount in situ hybridization probe sequences and plasmids are available at http://mamep.molgen.mpg.de, with accession numbers and sequences shown in Supplementary Table 10. The FISH probe accession codes can be found in Supplementary Table 10. Complete probe sequences are the property of Molecular Instruments. See the Supplementary Note for details on tutorials and additional user information for sc3D.

## Code availability

The code used to reproduce the analyses is indexed at https://github.com/GuignardLab/sc3D for the sc3D 3D reconstruction and https://github.com/LuyiTian/Embryo_Slideseq_analysis for the E9.5 analysis. The 3D embryo can be visualized with sc3D-viewer by following the detailed instructions provided (https://github.com/GuignardLab/napari-sc3D-viewer). PuckCaller can be accessed at https://github.com/MacoskoLab/PuckCaller.

## References

[1] Arnold SJ, Robertson EJ (2009). Making a commitment: cell lineage allocation and axis patterning in the early mouse embryo. Nat. Rev. Mol. Cell Biol..

[2] Rivera-Pérez, J. A. & Hadjantonakis, A.-K. The dynamics of morphogenesis in the early mouse embryo. *Cold Spring Harb. Perspect. Biol.* **7**, a015867 (2014). 10.1101/cshperspect.a015867 PMC4277506 24968703

[3] Selleck MA, Stern CD (1991). Fate mapping and cell lineage analysis of Hensen’s node in the chick embryo. Development.

[4] Tam PP, Behringer RR (1997). Mouse gastrulation: the formation of a mammalian body plan. Mech. Dev..

[5] Tam PPL, Loebel DAF (2007). Gene function in mouse embryogenesis: get set for gastrulation. Nat. Rev. Genet..

[6] Bachvarova RF (1999). Establishment of anterior-posterior polarity in avian embryos. Curr. Opin. Genet. Dev..

[7] Beddington RS, Robertson EJ (1999). Axis development and early asymmetry in mammals. Cell.

[8] Schier AF (2001). Axis formation and patterning in zebrafish. Curr. Opin. Genet. Dev..

[9] Zernicka-Goetz M (2002). Patterning of the embryo: the first spatial decisions in the life of a mouse. Development.

[10] Chapman DL, Agulnik I, Hancock S, Silver LM, Papaioannou VE (1996). *Tbx6*, a mouse T-Box gene implicated in paraxial mesoderm formation at gastrulation. Dev. Biol..

[11] Chapman DL, Papaioannou VE (1998). Three neural tubes in mouse embryos with mutations in the T-box gene *Tbx6*. Nature.

[12] Dickinson ME (2016). High-throughput discovery of novel developmental phenotypes. Nature.

[13] Harvey RP (2002). Patterning the vertebrate heart. Nat. Rev. Genet..

[14] Meehan TF (2017). Disease model discovery from 3,328 gene knockouts by The International Mouse Phenotyping Consortium. Nat. Genet..

[15] Copp AJ, Greene NDE, Murdoch JN (2003). The genetic basis of mammalian neurulation. Nat. Rev. Genet..

[16] Dessaud E, McMahon AP, Briscoe J (2008). Pattern formation in the vertebrate neural tube: a sonic hedgehog morphogen-regulated transcriptional network. Development.

[17] Roelink H (1995). Floor plate and motor neuron induction by different concentrations of the amino-terminal cleavage product of sonic hedgehog autoproteolysis. Cell.

[18] Simeone A, Acampora D, Gulisano M, Stornaiuolo A, Boncinelli E (1992). Nested expression domains of four homeobox genes in developing rostral brain. Nature.

[19] Sporle R, Schughart K (1997). Neural tube morphogenesis. Curr. Opin. Genet. Dev..

[20] Wilson L, Maden M (2005). The mechanisms of dorsoventral patterning in the vertebrate neural tube. Dev. Biol..

[21] Wurst W, Bally-Cuif L (2001). Neural plate patterning: upstream and downstream of the isthmic organizer. Nat. Rev. Neurosci..

[22] Argelaguet R (2019). Multi-omics profiling of mouse gastrulation at single-cell resolution. Nature.

[23] Cao J (2019). The single-cell transcriptional landscape of mammalian organogenesis. Nature.

[24] Chan MM (2019). Molecular recording of mammalian embryogenesis. Nature.

[25] Farrell, J. A. Single-cell reconstruction of developmental trajectories during zebrafish embryogenesis. *Science* **360**, eaar3131 (2018). 10.1126/science.aar3131 PMC6247916 29700225

[26] Grosswendt S (2020). Epigenetic regulator function through mouse gastrulation. Nature.

[27] Ibarra-Soria X (2018). Defining murine organogenesis at single-cell resolution reveals a role for the leukotriene pathway in regulating blood progenitor formation. Nat. Cell Biol..

[28] Tambalo, M., Mitter, R. & Wilkinson, D. G. A single cell transcriptome atlas of the developing zebrafish hindbrain. *Development* **147**, dev184143 (2020). 10.1242/dev.184143 PMC7097387 32094115

[29] Delile, J. et al. Single cell transcriptomics reveals spatial and temporal dynamics of gene expression in the developing mouse spinal cord. *Development* **146**, dev173807 (2019). 10.1242/dev.173807 PMC6602353 30846445

[30] Lohoff, T. et al. Integration of spatial and single-cell transcriptomic data elucidates mouse organogenesis. *Nat. Biotechnol.* **40**, 74–85 (2022). 10.1038/s41587-021-01006-2 PMC8763645 34489600

[31] McDole K (2018). In toto imaging and reconstruction of post-implantation mouse development at the single-cell level. Cell.

[32] Peng G (2019). Molecular architecture of lineage allocation and tissue organization in early mouse embryo. Nature.

[33] Rodriques SG (2019). Slide-seq: a scalable technology for measuring genome-wide expression at high spatial resolution. Science.

[34] Soldatov, R. et al. Spatiotemporal structure of cell fate decisions in murine neural crest. *Science* **364**, eaas9536 (2019). 10.1126/science.aas9536 31171666

[35] Srivatsan SR (2021). Embryo-scale, single-cell spatial transcriptomics. Science.

[36] van den Brink SC (2020). Single-cell and spatial transcriptomics reveal somitogenesis in gastruloids. Nature.

[37] Chen A (2022). Spatiotemporal transcriptomic atlas of mouse organogenesis using DNA nanoball-patterned arrays. Cell.

[38] Wang M (2022). High-resolution 3D spatiotemporal transcriptomic maps of developing *Drosophila* embryos and larvae. Dev. Cell.

[39] Stickels RR (2021). Highly sensitive spatial transcriptomics at near-cellular resolution with Slide-seqV2. Nat. Biotechnol..

[40] Zeira R, Land M, Strzalkowski A, Raphael BJ (2022). Alignment and integration of spatial transcriptomics data. Nat. Methods.

[41] Dono R (1993). The murine cripto gene: expression during mesoderm induction and early heart morphogenesis. Development.

[42] Fujii M (2017). *Sfrp5* identifies murine cardiac progenitors for all myocardial structures except for the right ventricle. Nat. Commun..

[43] Tanaka M, Chen Z, Bartunkova S, Yamasaki N, Izumo S (1999). The cardiac homeobox gene *Csx/Nkx2.5* lies genetically upstream of multiple genes essential for heart development. Development.

[44] Dworkin S, Jane SM (2013). Novel mechanisms that pattern and shape the midbrain–hindbrain boundary. Cell. Mol. Life Sci..

[45] Massarwa R, Ray HJ, Niswander L (2014). Morphogenetic movements in the neural plate and neural tube: mouse. Wiley Interdiscip. Rev. Dev. Biol..

[46] Millet S (1999). A role for *Gbx2* in repression of *Otx2* and positioning the mid/hindbrain organizer. Nature.

[47] Raible F, Brand M (2004). Divide et Impera—the midbrain–hindbrain boundary and its organizer. Trends Neurosci..

[48] Ishibashi M, McMahon AP (2002). A sonic hedgehog-dependent signaling relay regulates growth of diencephalic and mesencephalic primordia in the early mouse embryo. Development.

[49] Hettige NC, Ernst C (2019). *FOXG1* dose in brain development. Front Pediatr..

[50] Parish EV, Mason JO, Price DJ (2016). Expression of *Barhl2* and its relationship with Pax6 expression in the forebrain of the mouse embryo. BMC Neurosci..

[51] Shimamura K, Rubenstein JL (1997). Inductive interactions direct early regionalization of the mouse forebrain. Development.

[52] La Manno G (2018). RNA velocity of single cells. Nature.

[53] Broccoli V, Boncinelli E, Wurst W (1999). The caudal limit of *Otx2* expression positions the isthmic organizer. Nature.

[54] Rash BG, Grove EA (2011). *Shh* and *Gli3* regulate formation of the telencephalic–diencephalic junction and suppress an isthmus-like signaling source in the forebrain. Dev. Biol..

[55] Papachristou P, Dyberg C, Lindqvist M, Horn Z, Ringstedt T (2014). Transgenic increase of Wnt7b in neural progenitor cells decreases expression of T-domain transcription factors and impairs neuronal differentiation. Brain Res..

[56] Martinez-Ferre A, Navarro-Garberi M, Bueno C, Martinez S (2013). Wnt signal specifies the intrathalamic limit and its organizer properties by regulating Shh induction in the alar plate. J. Neurosci..

[57] Hiraga K (2020). Redundant type II cadherins define neuroepithelial cell states for cytoarchitectonic robustness. Commun. Biol..

[58] Chapman DL, Cooper-Morgan A, Harrelson Z, Papaioannou VE (2003). Critical role for *Tbx6* in mesoderm specification in the mouse embryo. Mech. Dev..

[59] Forlani S, Lawson KA, Deschamps J (2003). Acquisition of Hox codes during gastrulation and axial elongation in the mouse embryo. Development.

[60] Koch F (2017). Antagonistic activities of *Sox2* and *Brachyury* control the fate choice of neuro-mesodermal progenitors. Dev. Cell.

[61] Qiu X (2017). Reversed graph embedding resolves complex single-cell trajectories. Nat. Methods.

[62] Briggs, J. A. et al. The dynamics of gene expression in vertebrate embryogenesis at single-cell resolution. *Science* **360**, eaar5780 (2018). 10.1126/science.aar5780 PMC6038144 29700227

[63] Briscoe J, Ericson J (1999). The specification of neuronal identity by graded Sonic Hedgehog signalling. Semin. Cell Dev. Biol..

[64] Briscoe J, Pierani A, Jessell TM, Ericson J (2000). A homeodomain protein code specifies progenitor cell identity and neuronal fate in the ventral neural tube. Cell.

[65] Noordermeer D, Duboule D (2013). Chromatin architectures and *Hox* gene collinearity. Curr. Top. Dev. Biol..

[66] Zhu K, Spaink HP, Durston AJ (2017). Collinear Hox-Hox interactions are involved in patterning the vertebrate anteroposterior (A-P) axis. PLoS ONE.

[67] Takemoto T (2011). Tbx6-dependent *Sox2* regulation determines neural or mesodermal fate in axial stem cells. Nature.

[68] Andergassen D, Smith ZD, Kretzmer H, Rinn JL, Meissner A (2021). Diverse epigenetic mechanisms maintain parental imprints within the embryonic and extraembryonic lineages. Dev. Cell.

[69] Asimi V (2022). Hijacking of transcriptional condensates by endogenous retroviruses. Nat. Genet..

[70] Smith ZD (2017). Epigenetic restriction of extraembryonic lineages mirrors the somatic transition to cancer. Nature.

[71] Basch ML, Bronner-Fraser M (2006). Neural crest inducing signals. Adv. Exp. Med. Biol..

[72] Kwang SJ (2002). *Msx2* is an immediate downstream effector of *Pax3* in the development of the murine cardiac neural crest. Development.

[73] Pla P, Monsoro-Burq AH (2018). The neural border: induction, specification and maturation of the territory that generates neural crest cells. Dev. Biol..

[74] Sakai D, Wakamatsu Y (2005). Regulatory mechanisms for neural crest formation. Cells Tissues Organs.

[75] Sandberg M, Källström M, Muhr J (2005). Sox21 promotes the progression of vertebrate neurogenesis. Nat. Neurosci..

[76] Sofroniew, N. et al. napari: a multi-dimensional image viewer for Python (pre-release 0.4.17rc8). *Zenodo*10.5281/zenodo.7276432 (2022).

[77] Moncada R (2020). Integrating microarray-based spatial transcriptomics and single-cell RNA-seq reveals tissue architecture in pancreatic ductal adenocarcinomas. Nat. Biotechnol..

[78] Ji AL (2020). Multimodal analysis of composition and spatial architecture in human squamous cell carcinoma. Cell.

[79] Berglund E (2018). Spatial maps of prostate cancer transcriptomes reveal an unexplored landscape of heterogeneity. Nat. Commun..

[80] Veenvliet, J. V. Mouse embryonic stem cells self-organize into trunk-like structures with neural tube and somites. *Science* **370**, eaba4937 (2020). 10.1126/science.aba4937 33303587

[81] Hao Y (2021). Integrated analysis of multimodal single-cell data. Cell.

[82] Cable DM (2022). Robust decomposition of cell type mixtures in spatial transcriptomics. Nat. Biotechnol..

[83] Robinson MD, McCarthy DJ, Smyth GK (2010). edgeR: a Bioconductor package for differential expression analysis of digital gene expression data. Bioinformatics.

[84] Bergen V, Lange M, Peidli S, Wolf FA, Theis FJ (2020). Generalizing RNA velocity to transient cell states through dynamical modeling. Nat. Biotechnol..

[85] Korsunsky I (2019). Fast, sensitive and accurate integration of single-cell data with Harmony. Nat. Methods.

[86] Chen Y, Lun ATL, Smyth GK (2016). From reads to genes to pathways: differential expression analysis of RNA-Seq experiments using Rsubread and the edgeR quasi-likelihood pipeline. F1000Res..

[87] Street K (2018). Slingshot: cell lineage and pseudotime inference for single-cell transcriptomics. BMC Genomics.

[88] Sun S, Zhu J, Zhou X (2020). Statistical analysis of spatial expression patterns for spatially resolved transcriptomic studies. Nat. Methods.

[89] Mi H, Muruganujan A, Casagrande JT, Thomas PD (2013). Large-scale gene function analysis with the PANTHER classification system. Nat. Protoc..

[90] Haghverdi L, Lun ATL, Morgan MD, Marioni JC (2018). Batch effects in single-cell RNA-sequencing data are corrected by matching mutual nearest neighbors. Nat. Biotechnol..

