# Supplementary material for: Spatiotemporal transcriptomic maps of whole mouse embryos at the onset of organogenesis
Source: Nat Genet. 2023 Jul 6;55(7):1176–85. doi: 10.1038/s41588-023-01435-6 (PMC10335937; doi:10.1038/s41588-023-01435-6)
Supplement: Supplementary file 1 — Supplementary Figs. 1 and 2, Methods and Notes on the available data visualization. [file 41588_2023_1435_MOESM1_ESM.pdf]

nature genetics

Article

https://doi.org/10.1038/s41588-023-01435-6

Spatiotemporal transcriptomic maps of whole mouse embryos at the onset of organogenesis

In the format provided by the authors and unedited

Supplementary information

This file contains the following information:

Supplementary Figure 1
Supplementary Figure 2
Methods
Supplementary note for visualization of datasets

3D reconstruction E8.5 embryo and embryo 2

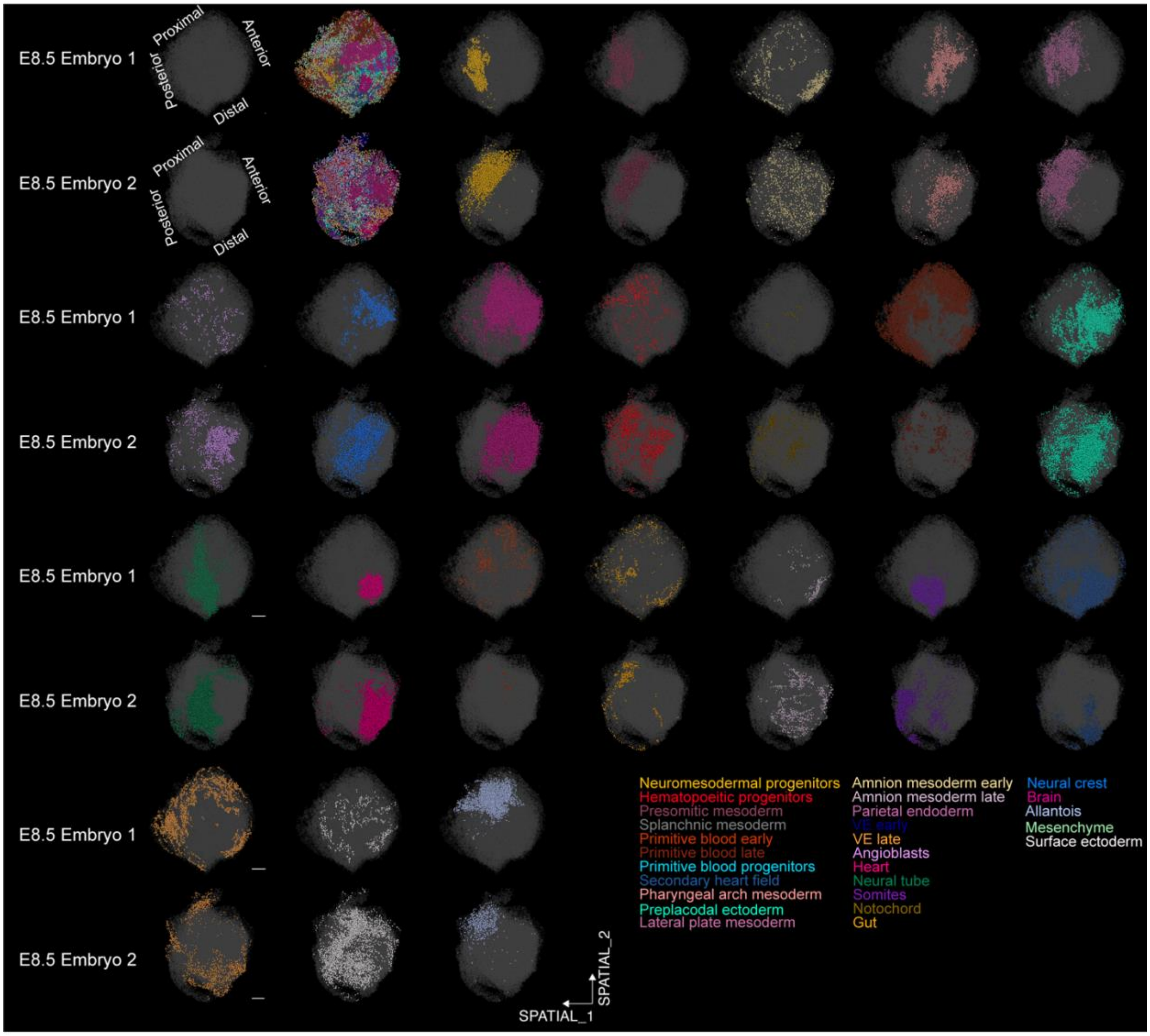

Supplementary Figure 1: **Spatial projection of the cell states in the two E8.5 stage 3D virtual embryos.** **a.** Each dot denotes a bead. Each color denotes a cell state. Scale bar is 200 μm.

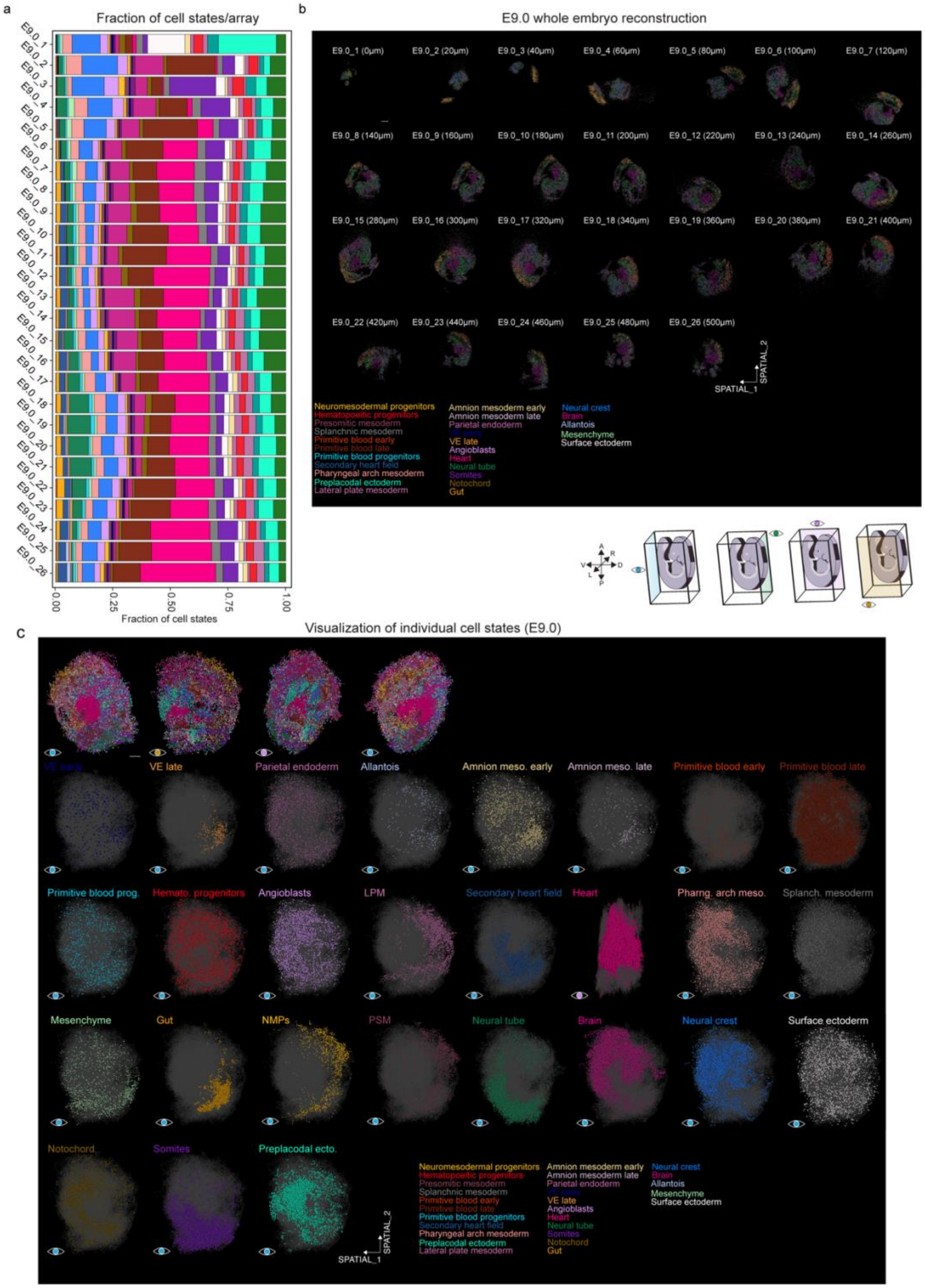

Supplementary Figure 2: **Slide-seq profiling of E9.0 stage embryos. a.** Cell state distribution of the annotated clusters in the individual arrays of the E9.0 stage whole embryo. Each color denotes a cell state. Annotation can be found in the next panel. **b.** Spatial plot of the cell states in the individual arrays of the E9.0 stage embryos. Each dot corresponds to a bead. Each color denotes a cell state. Scale bar is 200 μm. **c.** Spatial projection of the E9.0 stage 3D virtual embryo. Each dot corresponds to a bead. Each color denotes a cell state. Scale bar is 200 μm. A, anterior; P, posterior; L, left; R, right; D, dorsal; V, ventral.

Methods

Whole-embryo conventional *in situ* hybridization

We used plasmids from the MAMEP database for RNA probe synthesis (**http://mamep.molgen.mpg.de**) (Supplementary Table 10). PCR amplified the probe sequence and *in vitro* transcribed using DIG RNA Labeling Kit (SP6/T7) (*Roche*) and T3-RNA-Polymerase (*Roche*). The dissected embryos were fixed overnight in 4% PFA, transferred into 100% methanol by a methanol series (25%, 50%, 75%, 100%) and de-hydrated overnight in 100% methanol. Before hybridization, embryos were re-hydrated by a reverse methanol series and incubated in 6% hydrogen peroxide for 20min, followed by 10 μg/ml Proteinase K (EO0491, Thermo) treatment for 10 min. After a 20 min fixation in 0.2% Glutamine/4% PFA, embryos were incubated in Hybridization buffer (for detailed information about reagent compositions, *see* [7]) for 15 min at RT and an additional 2 h at 68°C. After another 15 min at 68°C in fresh Hyb, embryos were incubated with the RNA probe (200 ng/ul, preheated for 13 min at 80°C) o/n at 68°C, followed by multiple washing steps with Hyb, Solution1 and Solution3T at 68°C and TBST at RT. Anti-DIG antibody (*Roche*) 0.2% v/v was pre-incubated with 1% v/v lamb serum/TBST (*Sigma*) for 1h at 4°C and subsequently diluted to 1:200 with 1% lamb serum. Embryos were blocked in 10% lamb serum/TBST for 2.5h at RT and before incubation with the antibody solution overnight at 4°C. The next day, embryos were washed several times in TBST with a final washing step o/n at 4°C and stained with BM Purple (*Roche*) until a specific signal was imaged with a Zeiss Discovery 745 V12 microscope equipped with a Leica DFC420 digital camera. Probes were a kind gift from Bernard Herrmann, Max Planck Institute for Molecular Genetics, Berlin, and can be found at MAMEP with the indicated accession number in Supplementary Table 10.

Sc3D: 3D reconstruction and identification of spatially variable genes in 3D

When doing spatial single-cell transcriptomics, beads are recorded from arrays. Beads are placed on a 2D matrix where each bead is spaced by a given distance $\boldsymbol{x_{res}}$ (resp. $\boldsymbol{y_{res}}$) along the $\boldsymbol{x}$ (resp. $\boldsymbol{y}$) dimension (in our case, $\boldsymbol{x_{res} = y_{res} = 6\mu m}$). These distances define the $\boldsymbol{xy}$ resolution (or lateral resolution) of the slice or array. Then, consecutive arrays are spaced by a given distance $\boldsymbol{z_{res}}$ defining the $\boldsymbol{z}$ resolution (or axial resolution) of the dataset (in our case $\boldsymbol{z_{res} = 30\mu m}$).

Because the arrays are physically moved between their slicing and their acquisition, they are not acquired within the same frame (meaning that they are not aligned). To reconstruct a 3D representation of the single-cell transcriptomics of the acquired embryo and do complete 3D spatial analysis, it is necessary to align consecutive arrays to recover the spatial integrity of the specimen.

The following section will describe how this alignment was performed and how the beads were interpolated between arrays.

Notation

We define our dataset as a set of arrays $\boldsymbol{\mathcal{P} = \{P_i\}}$. The function $\boldsymbol{c_P}$ maps a array to its height coordinate $\boldsymbol{z_i}$: $\boldsymbol{c_P: P_i \in \mathcal{P} \rightarrow z_i} \in \mathbb{R}$. Each array $\boldsymbol{P_i}$ is itself a set of beads, $\boldsymbol{P_i = \{b_{ij}\}}$ and similarly to the arrays, the function $\boldsymbol{c_b}$ maps a bead $\boldsymbol{b_{ij}}$ to its $\boldsymbol{xy}$ coordinate within the array: $\boldsymbol{c_b: b_{ij} \in P_i \rightarrow (x, y)} \in \mathbb{R}^2$. From $\boldsymbol{c_P}$ and $\boldsymbol{c_b}$ we define the function $\boldsymbol{c: b_{ij} \in P_i \rightarrow (x, y, z)} \in \mathbb{R}$ which maps a bead to its 3D spatial coordinate. Note

that $\boldsymbol{c_P}$ defines a total order on the arrays. Let then $\boldsymbol{\mathcal{P}}$ be ordered such that $\forall \boldsymbol{i}, \boldsymbol{j},\ \boldsymbol{P_i} < \boldsymbol{P_j} \Leftrightarrow \boldsymbol{c_P}(\boldsymbol{P_i}) < \boldsymbol{c_P}(\boldsymbol{P_j})$.

Moreover, using the previously described analysis, we can associate each bead to the tissue it most likely belongs to, the function $\boldsymbol{T}: \boldsymbol{b_{ij}} \in \boldsymbol{P_i} \rightarrow \boldsymbol{t} \in \boldsymbol{\mathcal{T}}$ where $\boldsymbol{t} \in \boldsymbol{\mathcal{T}}$ maps each bead to a unique identifier for the tissue it belongs to. $\boldsymbol{\mathcal{T}}$ is the set of possible tissues previously identified. Similarly, to each bead is associated a value for each given gene analyzed in the dataset. This value is correlated to the level of expression of said gene for said bead. Given a gene $\boldsymbol{g}$, we define the function $\boldsymbol{E_g}: \boldsymbol{b_{ij}} \in \boldsymbol{P_i} \rightarrow \boldsymbol{e} \in \mathbb{R}$ which maps the value $\boldsymbol{e}$ of expression of the gene $\boldsymbol{g}$ to the bead $\boldsymbol{b_{ij}}$.

Pre-processing and removing the remaining outliers

Before aligning the arrays, removing beads that are likely to be noise is possible. Within a given tissue, noisy beads were detected as the beads that were spatially further away from their neighbors than the normal distribution of the spatial distances between beads. To assess the normal distribution of spatial distances between beads of a given tissue, we first computed the distance between any given bead and its closest bead from the same tissue type. We then analyzed the distribution of these distances by fitting a gaussian mixture model, with several components equal to $\boldsymbol{n_{components}}$ (in our case we used $\boldsymbol{n_{components}} = \boldsymbol{3}$). When using 3 components, the $\boldsymbol{1^{st}}$ and $\boldsymbol{2^{nd}}$ components are assumed to be accurate. The $\boldsymbol{3^{rd}}$ component, with the higher mean, is the distribution of distances of noisy beads. We then discarded all the beads that had a distance to their closest neighbor from the same tissue which had a probability of belonging to the first or second component were lower than $\boldsymbol{th_{gmm}}$ (in our case, we used $\boldsymbol{th_{gmm}} = \boldsymbol{0.6}\%$). This pre-processing step is not mandatory, but it can help get more accurate results. The value might vary depending on the sample and tissue analyzed.

Aligning the arrays

As previously mentioned, consecutive arrays do not live within the same frame due to the nature of the acquisition process. They are therefore not spatially comparable.

To align the arrays and register them onto the same frame, we first chose our first array in $\boldsymbol{\mathcal{P}}$, $\boldsymbol{P_0}$, as the reference array. We then registered each following slide to its preceding one: $\boldsymbol{P_1}$ is registered onto $\boldsymbol{P_0}$, $\boldsymbol{P_{i+1}}$ is registered onto $\boldsymbol{P_i}$ and so on. Ultimately, we can compose all the transformations together to register any array onto the first array. To compute the transformation necessary to register two consecutive arrays, we first performed a coarse grain alignment using the center of mass of a subset of the different tissue types. We then refined the alignment by pairing beads across the arrays and by aligning them.

Coarse grain alignment

We first chose a subset of tissues $\boldsymbol{\mathcal{L}} \subset \boldsymbol{\mathcal{T}}$ that is spatially localized (heart tube precursor beads or somite precursor beads). We then discarded (only for the coarse grain alignment) tissues that were spread in space (for example, blood precursor beads). The tissues that were discarded include blood-related cell states such as, hematopoietic progenitors, primitive blood late, angioblasts, primitive blood progenitors; extra-

embryonic cell states such as, visceral endoderm late, amnion mesoderm late, amnion mesoderm early, visceral endoderm early; and surface ectoderm, primordial germ cells.

Then we computed the alignment transformation (, registering the array $\boldsymbol{P_j}$ onto the array $\boldsymbol{P_i}$) as the rigid transformation (translation plus rotation) that minimizes the sum of the squared distances between corresponding tissue types center of mass:

$$\boldsymbol{r}^*_{i\leftarrow j} \quad = \quad \arg\max_{r\in\mathcal{R}}\left\{\sum_{t\in\mathcal{L}} \left\|\boldsymbol{COM_i(t)} - \boldsymbol{r}\left[\boldsymbol{COM_j(t)}\right]\right\|_2\right\}$$

(1)

Where $\boldsymbol{COM_i(t)}$ is the position of the center of mass of the tissue $\boldsymbol{t}$ in the array $\boldsymbol{i}$, $\|\cdot\|_2$ is the L2 norm and $\boldsymbol{\mathcal{R}}$ is the set of all rigid transformations. $\boldsymbol{r}\left[\boldsymbol{COM_j(t)}\right]$ is therefore the position of the center of mass of the tissue $\boldsymbol{t}$ in the array $\boldsymbol{j}$ after applying the rigid transformation $\boldsymbol{r}$.

We then applied the composition of the necessary transformations to register all arrays onto the first array. For example, to register the array $\boldsymbol{j}$ onto the array $\boldsymbol{0}$, we applied the transformation $\boldsymbol{r}^*_{0\leftarrow j} = \boldsymbol{r}^*_{0\leftarrow 1} \circ \boldsymbol{r}^*_{1\leftarrow 2} \circ \cdots \circ \boldsymbol{r}^*_{j-1\leftarrow j}$.

Alignment refinement

To refine the alignment, we then paired beads from consecutive arrays. Only beads from the same tissue types could be paired. The pairing was the one that minimizes the sum of the distances between paired beads (using the solution of the linear sum assignment optimization). The distances were computed after applying the coarse grain transformation. From this pairing, as previously, we computed the rigid transformation $\boldsymbol{R}^*_{i\leftarrow j}$ that minimizes the sum of the squares of the distances between the paired beads (see eq. (1)).

Spatial differential expression

The goal here was to score genes on whether they were locally expressed or not within a given tissue. To do so, we quantified if the set of genes that are considered expressing are spatially positioned next to each other. The metric we decided to use is based on the fact that the average degree of a graph (or network) is related to the average degree of that graph with randomly removed nodes. If a graph $\boldsymbol{G}$ has a density of $\boldsymbol{d(G) = k}$, then if a fraction $\boldsymbol{f}$ of nodes are removed randomly, building the new graph $\boldsymbol{G'}$, the density of $\boldsymbol{G'}$ is $\boldsymbol{d(G') \sim k(1-f)}$. But, if the nodes are not distributed randomly but are rather spatially localised, then the density of the new graph will be higher than the expected value: $\boldsymbol{d(G') > k(1-f)}$.

Having the previous paragraph in mind, it means that the more a gene expression will be localized, the further away the density of the graph of expressing beads will be from the expected value of $\boldsymbol{k(1-f)}$

In the context of our study, $\boldsymbol{G = (V, E)}$ is the graph where the vertices are the beads with their spatial positions. Then, to build the set of edges $\boldsymbol{E}$ between the vertices we computed the Gabriel graph on $\boldsymbol{V}$.

Finally, the fraction of removed nodes ($\boldsymbol{f}$) is the fraction of the total number of beads in which beads are not expressing a given gene.

To compute $\boldsymbol{f}$ we first computed the expression threshold above which a bead is considered expressing. The threshold was computed independently for each gene-tissue pair as the value that splits the distribution of expression values for each gene within each tissue into two classes (expressing and not expressing beads). For that we used the Otsu method [11] which splits a distribution into two classes such that the intra-class variance is minimum (and therefore maximizing the inter-class variance). For each gene $\boldsymbol{g}$, we therefore have computed a threshold $\boldsymbol{O_{th}(g)}$.

Having split the beads in two separate classes, we create the new graph $\boldsymbol{G'_g = (V'_g, E'_g)}$ where $\boldsymbol{V'_g = \{v \in V \mid O_{th}(g) < ge(g, v)\}}$ with $\boldsymbol{ge(g, v)}$ is the expression of the gene $\boldsymbol{g}$ of the vertice (or bead) $\boldsymbol{v}$. The set of edges $\boldsymbol{E'_g}$ is then the set of edges included in $\boldsymbol{E_g}$ such that both vertices of the edges in $\boldsymbol{E'_g}$ are in the set of expressing beads ($\boldsymbol{V'_g}$): $\boldsymbol{E'_g = \{(v_1, v_2) \in E_g \mid (v_1, v_2) \in V'^2_g\}}$. $\boldsymbol{G'_g}$ is the graph of connections between beads expressing a given gene $\boldsymbol{g}$.

We then computed the densities of $\boldsymbol{G_g}$ and $\boldsymbol{G'_g}$ as followed:

$$\boldsymbol{d(G_g) = \frac{2.|E_g|}{|V_g|(|V_g| - 1)}}.$$

The fraction of removed nodes is computed as $\boldsymbol{f_g = 1 - \frac{|V'_g|}{|V_g|}}$. This value can be seen as the ratio of volume expressing a given gene over the total volume. We also normalised the values so they are comparable across different genes and tissues, we are therefore looking at $\boldsymbol{f_g}$ (which is already normalised) and $\boldsymbol{d_g = \frac{d(G'_g)}{d(G_g)}}$.

We can then look at $\boldsymbol{d_g}$ against $\boldsymbol{f_g}$ knowing that $\boldsymbol{d(G'_g) \sim d(G_g)f_g}$ and therefore $\boldsymbol{d_g \sim f_g}$. Because of the potential noise of the dataset, instead of taking the theoretical value, we computed the linear regression between the distributions $\boldsymbol{d_g}$ and $\boldsymbol{f_g}$ for each gene within a given tissue. Then, for each gene, the distance to the linear regression is calculated which is the score for spatially expressed genes. The higher the score, the further the gene is from the expected value, and therefore the further it is from being randomly expressed in space, meaning that it is locally expressed.

Comparison with PASTE:

We compared the results of our inter-array registration algorithm to the ones of PASTE described in the publication (PMID: 35577957). The main difference between sc3D registration method and PASTE's is that our method relies heavily on the pre-annotation of the tissues of the dataset. PASTE on the other hand only uses RNAI counts per bead and finds the transformation that minimizes the difference between paired beads across arrays. For that reason PASTE allows to perform array registration without having to pre-process the data as much as sc3D but sc3D is usually significantly faster since the optimization part is done at a higher level.

To compare PASTE to sc3D we used both our dataset and the human dorsolateral prefrontal cortex (DLPFC) from that was used in the PASTE article. When we ran PASTE on the DLPFC dataset we used the optimal parameters provided by the article. When we ran PASTE on our dataset, we tried different parameterization and kept the best. To compare our results to those of PASTE we used a similar metric than the one used in the PASTE article for non-simulated datasets: the fraction of beads that have their closest bead in the neighboring arrays belonging to the same tissue. To compute our accuracy metric, we first compute a pairing between beads of consecutive arrays. The pairing is a spatial proximity pairing where two beads are paired if they are closed spatially. Effectively we computed the optimal pairing $\pi^\star$ over the set of all the possible pairings Π that minimises the sum of the distances between paired beads:

$$\pi^\star = \arg\min_{\pi\in\Pi} \sum_{(b_{i,j},b_{i+1,k})\in\pi} \left\| b_{i,j}, b_{i+1,k} \right\|_2$$

where $\left(b_{i,j}, b_{i+1,k}\right) \in \pi$ are two paired beads in π. To do so we used the Hungarian algorithm that gives an optimal result. Given this pairing, we can now count the percentage of pairs that have the same tissue over the total number of pairs. The higher this percentage is, the better the alignment. Figures 2 and 3 show the accuracy of the alignment between our method and PASTE. Note that for the DLPFC the results are similar and that for the mouse dataset sc3D performs better. It is important to keep in mind that the parameterization that we found for PASTE on our dataset might not be the best even though we tried to optimize it.

Supplementary note for sc3D-Visualizer:

Registered 3D datasets for the two E8.5 replicate embryos and E9.0 embryo can be found in the link below:

E8.5_Embryo_2 -> https://doi.org/10.6084/m9.figshare.21695849.v1

E8.5_Embryo_1 -> https://figshare.com/s/1c29d867bc8b90d754d2

E9.0 -> https://doi.org/10.6084/m9.figshare.21695879.v1

Instructions to install and use sc3D-visualizer can be found in the link below:

https://github.com/GuignardLab/napari-sc3D-viewer

Supplementary information for CellXGene visualization:

CellXGene data visualizations include a spatial map of cell states, spatial gene expression, and cell cycle stages.

*The links are not supported by Safari browser

https://cellxgene.cziscience.com/collections/d74b6979-efba-47cd-990a-9d80ccf29055/

The websites below provide tutorials on how to download and visualize the data.

https://github.com/chanzuckerberg/cellxgene-documentation/blob/main/README.md

https://github.com/chanzuckerberg/cellxgene-documentation/blob/main/portal/data-portal.md

https://github.com/chanzuckerberg/cellxgene-documentation/blob/main/explore-data/explorer-tutorials.md

To visualize cell states, navigate to the "Cell state" tab. Follow the tab labeled "Seurat clusters" for E9.5 brain sub-clustering. Annotations for relevant Seurat_clusters: 0, forebrain/presumptive telencephalon; 1, hindbrain; 2, somites, 4, heart; 5, premigratory neural crest; 6, Allantois; 7, posterior neural tube; 8, 10, primitive blood late; 9, tail bud progenitors; 11, cardiac neural crest; 12, pharynx progenitors; 13, branchial arch; 15, spinal cord; 16, secondary heart field; 17, future nephric duct; 18, frontonasal process; 21, mid/hindbrain/future isthmus; 22, notochord; 23, diencephalon/midbrain ventral; 24, hindlimb mesenchyme; 26, hindgut; 27, diencephalon/midbrain dorsal; 29, biliary bud; 30, otic pit. See Supplementary Table 1 for array_IDs.
